# Improved Dung Beetle Optimizer–Tuned Convolutional–Recurrent Temporal Self-Attention Network for Multi-Horizon Traffic Speed Forecasting

**DOI:** 10.3390/s26196145

**Published:** 2026-09-28

**Authors:** Jiwu Tang, Linchao An, Haifeng Liu, Jiaze Wang

**Affiliations:** 1 College of Applied Technology, Dalian Ocean University, Dalian 116300, China; tangjiwu@dlou.edu.cn; 2 College of Mechanical and Power Engineering, Dalian Ocean University, Dalian 116023, China

**Keywords:** traffic-speed forecasting, multi-horizon forecasting, multi-location context, convolutional–recurrent network, temporal self-attention, bio-inspired hyperparameter optimization

## Abstract

Reliable traffic-speed forecasting requires local and temporal dependence to be modeled under consistent evaluation. This study presents an Improved Dung Beetle Optimizer (IDBO)-selected convolutional–recurrent temporal self-attention network for direct three-location forecasting. Separate models predict 5, 15, and 30 min speeds from synchronized 60 min histories in the Caltrans Performance Measurement System (PeMS) freeway and Harbin urban-road case studies. IDBO combines stagnation-triggered Lévy perturbation and adaptive weighting to select convolutional filters, recurrent units, and attention heads using a shared six-task validation objective. Chronological evaluation includes nine forecasting methods, five final runs, paired inference, matched-budget search comparisons, context controls, and ablations. The proposed configuration achieves the lowest three-location macro root mean square error (RMSE), mean absolute error, and denominator-bounded mean absolute percentage error (ε-MAPE, with ε = 5 km/h) among the nine benchmark methods in all six tasks. Relative to Transformer, RMSE reductions are 13.97–14.42% on PeMS and 12.79–13.08% on Harbin; reductions relative to its capacity-matched counterpart are 7.55–7.92% and 6.66–7.24%, respectively. All twelve paired comparisons remain significant after separate six-task Holm adjustments for the two comparators. IDBO achieves the lowest mean normalized search endpoint and area under the curve under the common query budget. The network uses 240,387 parameters and 2,804,672 analytical multiply–accumulate operations per sample, documenting the accuracy–complexity trade-off.

## 1. Introduction

Accurate short-term traffic-speed forecasting is a core analytical capability for proactive traffic management, dynamic route guidance, and corridor-level control [1]. At five-minute resolution, however, speed series combine recurrent daily structure with abrupt transitions and location-dependent variability, making reliable 5–30 min prediction a demanding multiscale sequence-learning problem.

Road conditions observed at one location are also shaped by the state of the surrounding corridor. Nearby-road impact analysis has shown that surrounding links can add useful information to traffic-speed prediction when their relationships are represented explicitly [2]. This motivates a compact corridor formulation in which concurrent neighboring speeds form ordered input channels and provide synchronized cross-location context.

Existing traffic-speed models range from statistical and kernel methods to recurrent, convolutional, and attention-based networks. Graph-based forecasting also combines multi-scale temporal transformations with dynamic road relationships [3]. The practical design problem is to combine local and sequential features and select their dimensions effectively. This study addresses that problem through an integrated forecasting architecture, a capacity-matched prediction comparison, and configuration searches under a common budget.

This study develops an Improved Dung Beetle Optimizer (IDBO)-tuned network that combines a convolutional neural network (CNN), long short-term memory (LSTM), and a temporal self-attention network (TSAN) for direct three-location traffic-speed forecasting. A one-dimensional CNN extracts local variation, LSTM preserves sequence order, and TSAN weights historical positions. IDBO selects the structural configuration through validation before inference. Separate models use synchronized 60 min histories to predict 5, 15, and 30 min speeds in the Caltrans Performance Measurement System (PeMS) freeway and Harbin urban-road case studies. The principal contributions are as follows:

1. A sequence-preserving CNN–LSTM–TSAN architecture combines local filtering, recurrent memory, and temporal weighting for synchronized three-location forecasts. The full historical sequence passes through convolution and recurrent processing, allowing temporal attention to weight states that retain both local variation and ordered dependence before the direct three-output head. Component removals, synchronized-context controls and a capacity-matched Transformer connect this design to its observed forecasting gains under the same evaluation protocol.
2. IDBO extends the standard Dung Beetle Optimizer (DBO) with stagnation-triggered Lévy perturbation and iteration-dependent adaptive weighting for bounded selection of convolutional filters, recurrent units, and attention heads. Common search spaces, decoding rules, candidate budgets, initialization seeds, and validation fitness allow the two updates to be assessed against random search, particle swarm optimization (PSO), standard DBO, and the two component-removal controls. The selected configuration has 64 convolutional filters, 128 recurrent–attention units, and four attention heads (F64/U128/A4). Section 4.4.4 evaluates the mean normalized endpoint, area under the curve (AUC), endpoint variation and full-grid selection gap while distinguishing query efficiency from cache-construction cost.
3. A controlled evaluation combines nine forecasting methods, five-run final reporting, task-level paired inference, matched-budget optimizer histories, context and component comparisons, robustness diagnostics, and analytical complexity. Error magnitude, signed bias and residual behavior complement root mean square error (RMSE), while the capacity-matched and coupled-width comparisons show how the forecasting gain relates to model configuration. Optimizer histories separately characterize selection quality under the common query budget.

The remainder of this paper is organized as follows. Section 2 reviews traffic forecasting and validation-driven hyperparameter optimization. Section 3 presents the forecasting architecture and offline IDBO procedure. Section 4 introduces the data and experimental design, reports comparative and controlled analyses, and discusses the findings. Section 5 concludes the paper.

## 2. Related Work

Traffic forecasting research has evolved from parsimonious time-series models to learning systems that represent nonlinear temporal and cross-location dependence. Recent long-horizon work strengthens this direction by modeling global–local, multiscale spatio-temporal correlations with adaptive graph structures [4]. Multistep traffic-speed studies also combine temporal histories with spatial context to model horizon-dependent behavior [5]. Across these settings, error typically grows with the prediction horizon, underscoring the need to evaluate several lead times rather than one favorable task.

Autoregressive integrated moving average (ARIMA) and vector autoregression (VAR) represent linear temporal or multivariate dependence, while support vector regression (SVR) introduces nonlinear kernel mapping. These models are comparatively interpretable and can remain competitive when data are limited, but fixed linear structure or shallow feature mappings may not fully capture abrupt traffic transitions. Persistence, VAR, and SVR are therefore retained in this study as substantive reference points.

Deep sequence models extend this capacity. LSTM mitigates vanishing gradients through gated memory [6]. Zhang et al. combine CNN, LSTM and gated recurrent units for highway traffic-flow prediction [7]. Cao et al. represent multi-view temporal characteristics as embeddable graphs in a traffic-speed prediction network [8]. Transformer-style self-attention connects separated historical positions [9], and Wu et al. combine graph convolution, multi-scale temporal convolution and attention-based multi-dimensional LSTM for network-scale forecasting [10]. Recent road-speed prediction also combines graph-convolutional spatial feature extraction with segment-recurrent temporal modeling [11]. These studies show that combining local, recurrent and attention representations is established practice. The present design retains a compact three-channel history through CNN and LSTM before temporal self-attention; the structural removals and matched-capacity comparison test the resulting configuration under a common protocol.

Graph-attention models represent dynamic road relationships across time scales [12], while self-attention and multigraph formulations learn adaptive spatio-temporal dependencies [13]. Context-aware knowledge graphs incorporate explicit road information [14], and graph-attention forecasting can prioritize features across larger networks [15]. Here, three synchronized location channels provide a compact context representation, and self-attention acts along time. Self-only, Three-site, and Shuffled-context comparisons examine the predictive value of additional-location histories within this monitored set.

Traffic-forecasting performance can depend on parameter optimization and adaptive model selection [16]. Tian and Li combine parabolic adaptive parameter substitution, Gaussian parameter substitution and a Lévy-flight escape strategy in a DBO variant [17]. Here, IDBO specifies a stagnation-triggered, leader-relative Lévy perturbation and iteration-dependent weighting for the three-variable traffic-network configuration search. The two removal controls examine these changes separately, and random search, the original PSO formulation [18] and standard DBO provide common-budget references. Section 3.1 defines the updates; Section 4.3.2 fixes the candidate space, initialization, evaluator and query budget.

## 3. Proposed Methodology

Figure 1 outlines the data-isolated workflow used to train and evaluate one model for each dataset–horizon task. Evaluation uses mean absolute error (MAE), root mean square error (RMSE), denominator-bounded mean absolute percentage error (ε-MAPE, with *ε* = 5 km/h), and signed mean error (ME).

**Figure 1:**
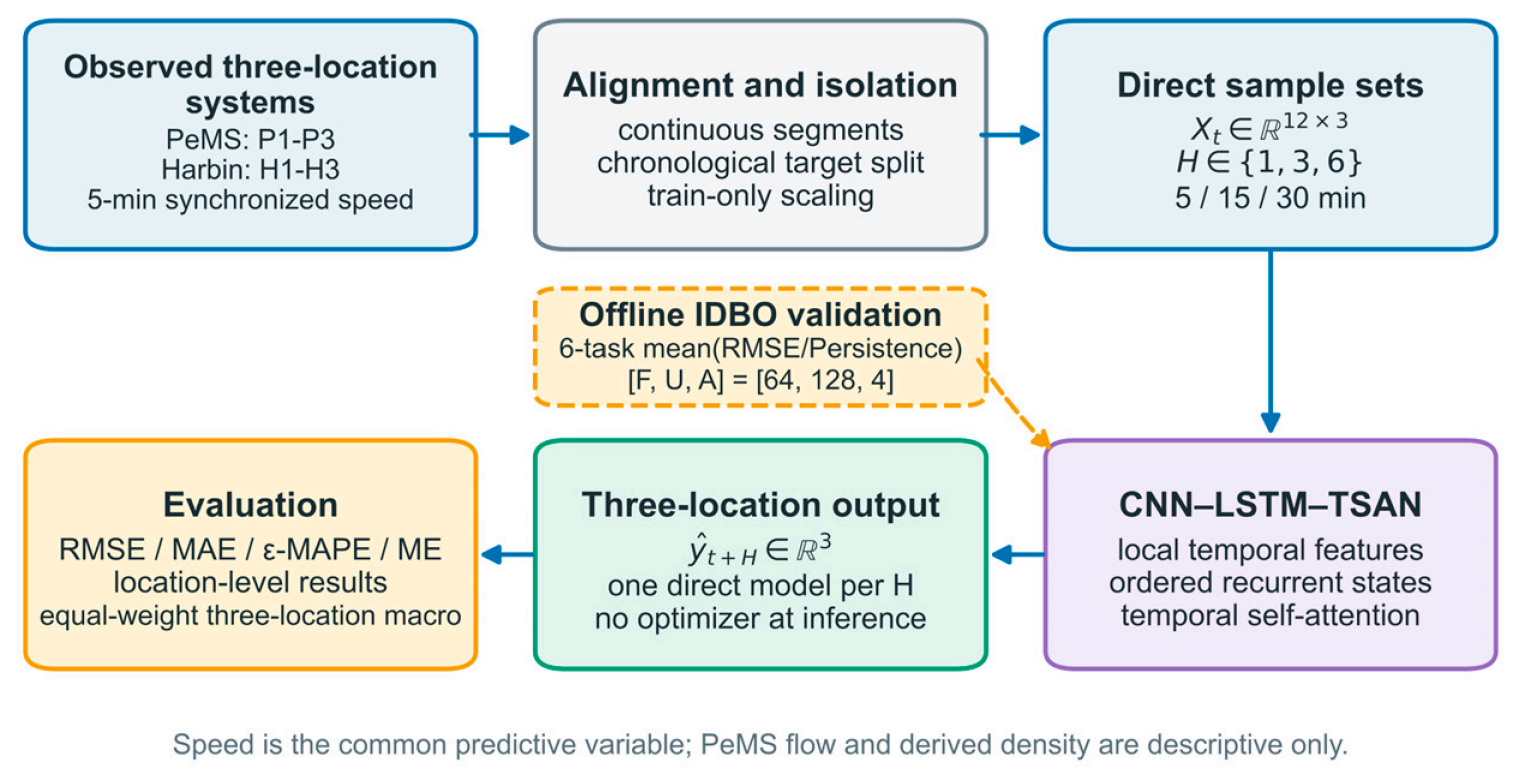
Independent three-location traffic-speed forecasting workflows for the Caltrans Performance Measurement System (PeMS) freeway and Harbin urban-road datasets at 5, 15, and 30 min horizons. *F*, *U*, and *A* denote convolutional filters, coupled recurrent–attention width, and attention heads, respectively.

For each dataset, the architecture maps a synchronized 12-step history to three direct speed estimates. Metrics average the location-level errors equally. Training and validation determine the shared configuration before separate task-specific fitting; test targets are reserved for final evaluation.

Within each dataset, samples are formed independently inside continuous segments to preserve chronological order (Figure 2).

**Figure 2:**
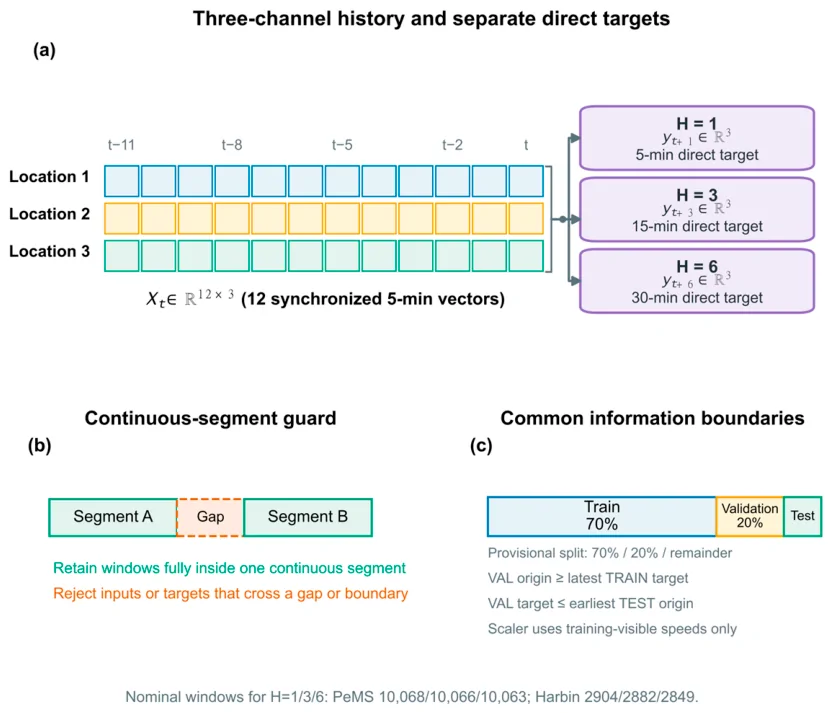
Gap-aware sample construction: (**a**) 12-step, three-channel histories and separate direct targets for 5, 15, and 30 min forecasts; (**b**) continuous-segment constraints; (**c**) chronological splits and common information boundaries across horizons. The bottom-row counts are nominal windows before target-completeness and common information-boundary filtering; Table 1 reports the final eligible counts.

**Table 1:** Dataset coverage, monitored locations, fields, historically filled entries, and nominal/eligible sample counts for each forecast horizon. Training and test memberships are fixed from nominal chronological boundaries. Validation additionally satisfies the dataset-wide information-availability rule across horizons. Imputation is reported as common timestamps followed by site-level cells in parentheses.

Dataset	Locations	Coverage	Aligned Time Slots/Segments/Historically Imputed Slots (Cells)	Fields	Horizon Step (min)	Samples Nominal/Eligible Train/Val/Test
PeMS	P1–P3	26 November 2021 00:05 –31 December 2021 00:00	10,080/1/0 (0)	Speed (km/h); descriptive only: Flow and derived density	1 (5)	10,068/10,062 7047/2007/1008
PeMS	P1–P3	26 November 2021 00:05 –31 December 2021 00:00	10,080/1/0 (0)	Speed (km/h); descriptive only: Flow and derived density	3 (15)	10,066/10,058 7046/2005/1007
PeMS	P1–P3	26 November 2021 00:05 –31 December 2021 00:00	10,080/1/0 (0)	Speed (km/h); descriptive only: Flow and derived density	6 (30)	10,063/10,053 7044/2002/1007
Harbin	H1–H3	10 May 2022 –20 May 2022	3036/11/19 (57)	Speed (km/h)	1 (5)	2904/2883 2032/573/278
Harbin	H1–H3	10 May 2022 –20 May 2022	3036/11/19 (57)	Speed (km/h)	3 (15)	2882/2860 2017/567/276
Harbin	H1–H3	10 May 2022 –20 May 2022	3036/11/19 (57)	Speed (km/h)	6 (30)	2849/2825 1994/558/273

Each history window $X_{t}\in R^{\left(12\times 3\right)}$ directly predicts $y_{t+H}\in R^{3}$. Each input window and its target lie within a single continuous segment; samples are ordered by target timestamp and partitioned into 70% training, 20% validation, and the remainder for testing. Scaling parameters are estimated from the training partition and applied unchanged to the validation and test partitions. Each horizon-specific target contains all three locations; the row colors in Figure 2 identify locations rather than forecast horizons.

To align information availability across the shared six-task selection, each dataset uses one latest training-target time and one earliest test-forecast origin across its three horizons. A provisional validation member is retained only if its forecast origin is at or after that training cutoff and its target is at or before that test-origin cutoff. The input ends at the forecast origin. Training and test memberships remain fixed; validation eligibility depends on timestamps rather than observed errors or model identity.

Figure 3 links offline hyperparameter selection to the forward graph used for each independently trained dataset–horizon task. Joint validation over the six tasks selects 64 convolutional filters, 128 recurrent–attention units, and four attention heads. The upper panel shows functional dependencies. In the cached evaluation of Algorithm 1, candidate training and validation populate the shared component matrix once; subsequent search proposals query these stored values without refitting candidate networks.

One-dimensional convolution preserves all 12 historical positions, the sequence-returning long short-term memory layer maps their ordered dependence, and temporal self-attention heads reweight historical positions before global pooling and the three-location output head. The symbols *F*, *U*, and *A* denote the number of convolutional filters, the recurrent hidden width, and the number of attention heads, respectively; *U* also sets the attention model dimension.

### 3.1. Improved Dung Beetle Optimizer (IDBO)

The Dung Beetle Optimizer (DBO), introduced by Xue and Shen [19], uses rolling, breeding, foraging, and stealing updates. Equations (1)–(8) define these standard stages; Equations (9)–(12) give the two IDBO additions.

#### 3.1.1. Rolling Ball Dung Beetle

The rolling group provides a broad position update. In unobstructed mode, the candidate moves with respect to its previous position and the current worst solution, as defined in Equations (1) and (2). Obstacle mode changes the direction through Equation (3).

(1) $$X_{i}\left(t+1\right)=X_{i}\left(t\right)+\alpha \times k\times X_{i}\left(t-1\right)+b\times \left|X_{i}^{t}-X^{W}\right|$$

(2) $$\Delta X=\left|X_{i}^{t}-X^{W}\right|$$

In Equations (1) and (2), t is the iteration index; $X_{i}\left(t\right)\in R^{D}$, $X^{W}$, and $\Delta X=|X_{i}\left(t\right)-X^{W}|$ denote candidate *i*, the current worst position, and their element-wise distance. The fixed coefficients are k=0.1 and b=0.3. For each unobstructed update, α=−1 if $u_{\alpha }<0.5$ and α=+1 otherwise, where $u_{\alpha }∼U\left(0,1\right)$. Every updated position is clipped to the search bounds.

For each rolling candidate, draw $u_{roll}∼U\left(0,1\right)$: use Equations (1) and (2) if $u_{roll}<0.9$ and Equation (3) otherwise. At the first update (t=1), set $X_{i}\left(0\right)=X_{i}\left(1\right)$ to the initialized candidate position.

(3) $$X_{i}\left(t+1\right)=X_{i}\left(t\right)+tan\left(\theta \right)\left|X_{i}\left(t\right)-X_{i}\left(t-1\right)\right|$$

The angle θ is drawn from [0,π] subject to a finite tangent; $\frac{\pi }{2}$ is therefore excluded. Angles 0 and π produce zero displacement, and any inadmissible draw is resampled before the update. The term $\left|X_{i}\left(t\right)-X_{i}\left(t-1\right)\right|$ is the magnitude of the previous candidate step.

#### 3.1.2. Breeding Dung Beetle

The breeding group searches a shrinking region around the current local best position. Equation (4) defines the region and Equation (5) generates the next breeding candidate.

(4) $$\{\begin{array}{c} {Lb}^{*}=\max\left(X^{*}\times \left(1-R\right),Lb\right)\\ {Ub}^{*}=\min\left(X^{*}\times \left(1+R\right),Ub\right) \end{array}$$

In Equation (4), $X^{*}$ is the current local best position, $R=1-\frac{t}{T_{max}}$, and $T_{max}$ is the maximum number of iterations. Lb and Ub are the lower and upper global bounds; ${Lb}^{*}$ and ${Ub}^{*}$ are the corresponding breeding-region bounds.

Equation (5) updates each breeding candidate within the current region. Because *R* decreases with *t*, the region contracts as the search proceeds; the resulting vector is clipped to the global bounds.

(5) $$B_{i}\left(t+1\right)=X^{*}+b_{1}\times \left(B_{i}\left(t\right)-{Lb}^{*}\right)+b_{2}\times \left(B_{i}\left(t\right)-{Ub}^{*}\right)$$

In Equation (5), $B_{i}\left(t\right)$ is breeding candidate *i* at iteration *t*, and $b_{1}$ and $b_{2}$ are independent *D*-dimensional vectors sampled component-wise from U(0,1).

#### 3.1.3. Foraging Dung Beetle

The foraging group searches around the current global best position. Equation (6) defines a time-varying foraging region, and Equation (7) updates the associated candidates.

(6) $$\{\begin{array}{c} {Lb}^{l}=\max\left(X^{l}\times \left(1-R\right),Lb\right)\\ {Ub}^{l}=\min\left(X^{l}\times \left(1+R\right),Ub\right) \end{array}$$

In Equation (6), $X^{l}$ is the current global best position; ${Lb}^{l}$ and ${Ub}^{l}$ are the lower and upper bounds of the foraging region. The decay factor R and global bounds Lb and Ub are defined as in Equation (4).

(7) $$X_{i}\left(t+1\right)=X_{i}\left(t\right)+C_{1}\times \left(X_{i}\left(t\right)-{Lb}^{l}\right)+C_{2}\times \left(X_{i}\left(t\right)-{Ub}^{l}\right)$$

In Equation (7), $X_{i}\left(t\right)$ is candidate *i*, $C_{1}∼N\left(0,1\right)$, and $C_{2}$ is a *D*-dimensional vector sampled component-wise from U(0,1). The updated position is clipped to [Lb,Ub].

#### 3.1.4. Stealing Dung Beetle

The stealing group exploits promising regions around the current best solutions. Its standard DBO position update is given in Equation (8).

(8) $$X_{i}\left(t+1\right)=X^{l}+S\times g\times \left(\left|X_{i}\left(t\right)-X^{*}\right|+\left|X_{i}\left(t\right)-X^{l}\right|\right)$$

In Equation (8), $X_{i}\left(t\right)$ is stealing candidate *i*; $X^{*}$ and $X^{l}$ denote the local and global best positions; $g∼N\left(0,I_{D}\right)$ is a *D*-dimensional Gaussian vector; and the standard DBO coefficient is S=0.5.

#### 3.1.5. Lévy Flight and Adaptive Search Weighting

IDBO modifies the stealing update with a uniformly scaled, heavy-tailed Mantegna step and an iteration-dependent search weight. Frequent short steps preserve local refinement, while occasional long steps can move a candidate outside its current neighborhood when the incumbent fitness stops improving.

Stagnation is defined operationally as no decrease in the best joint validation objective between two successive completed IDBO iterations. On the next iteration, Equation (9) replaces the standard stealing update for the stealing subgroup; any improvement resumes the standard cycle. This search trigger is distinct from the five-epoch early-stopping patience used when fitting a candidate network.

(9) $$X_{i}\left(t+1\right)=X^{l}+Levy\left(s\right)\times (X_{i}(t)-X^{l})\;\;\;\;\;\;\;\;\;\;\;\;+S\times g\times \left(\left|X_{i}\left(t\right)-X^{*}\right|+\left|X_{i}\left(t\right)-\omega \times X^{l}\right|\right)$$

The Lévy factor in Equation (9) is generated by the uniformly scaled Mantegna construction in Equations (10) and (11) [20]. All perturbed candidates are clipped to the bounded search space before integer decoding.

(10) $$Levy\left(s\right)=\frac{0.01\times x\times \eta }{{\left|y\right|}^{\frac{1}{\beta }}}$$

In Equations (10) and (11), η∼U(0,1) supplies independent uniform scaling of the Mantegna ratio, $x∼N\left(0,{\sigma_{x}}^{2}\right)$, y∼N(0,1), and the stability index is fixed at β=1.5. The argument s indexes the generated step and is unrelated to the final-training seed r. The symbol β is distinct from the standard DBO coefficient S=0.5.

(11) $$\begin{array}{c} x∼N\left(0,{\sigma_{x}}^{2}\right),y∼N\left(0,1\right)\\ \sigma_{x}={\left(\frac{\Gamma \left(1+\beta \right)sin\left(\frac{\pi \beta }{2}\right)}{\Gamma \left(\frac{1+\beta }{2}\right)\times \beta \times 2^{\frac{\beta -1}{2}}}\right)}^{\frac{1}{\beta }} \end{array}$$

Equation (12) defines $\omega \left(t\right)=tanh\left(2\times \left(1-\frac{t}{T_{max}}\right)\right)$, which decreases to zero over the search. In Equation (9), the weight modifies $X^{l}$ inside the state-dependent absolute difference. Although the adaptive weight decreases over the search, the additive Lévy displacement in Equation (9) allows the realized displacement to vary non-monotonically across iterations. In the matched ablations, w/o Lévy sets Levy(s)=0 and w/o adaptive weighting sets ω=1. All population updates read an immutable iteration-start state: the local best is the minimum of the ten current candidates, the global best is the all-time incumbent, and the worst position is the current population maximum. Duplicate configurations are replaced by the first unused index in a shared coprime-stride scan of the 1740 configuration IDs; the coordinate mapping, tie-breaking rules and update order are specified below.

The configuration order is ascending in *F*, then *U*, then *A*, with *A* varying fastest. The three zero-based grid indices are normalized by 14, 28 and 3. Coordinates are clipped to the unit cube and rounded to the nearest grid indices with ties to even; the configuration ID is four times the sum of 29 times the filter index and the width index, plus the head index. Duplicate IDs are replaced by the first unused ID in a seeded coprime-stride scan of the 1740 IDs. Each scan uses one shared start and stride indexed by the search seed, proposal number and raw duplicate ID; the method name is excluded from this key. Only the resulting unused ID is evaluated and counted as one query; scanning previously used IDs does not add objective evaluations. Best-position ties choose the smallest ID and worst-position ties the largest. The 2 rolling, 2 breeding, 3 foraging and 3 stealing candidates use one immutable iteration-start snapshot; duplicate repairs are then committed in candidate order. Lévy draws are independent in each of the three coordinates.

(12) $$\omega \left(t\right)=tanh\left(2\left(1-\frac{t}{T_{max}}\right)\right)$$

### 3.2. Sequence-Preserving Convolutional Encoder

The convolutional encoder applies *F* same-padded, kernel-three filters with rectified linear unit (ReLU) activation along time. The three locations are input channels, and all 12 historical positions pass to the recurrent layer.

(13) $$g_{j}^{l}=\sum_{i}Conv1D\left(x_{i}^{l-1},\omega_{i,j}^{l}\right)+b_{j}^{l}$$

In Equation (13), l indexes the layer, i an input channel, and j an output filter. The terms $x_{i}^{l-1}$, ${\omega_{i,j}}^{l}$ and ${b_{j}}^{l}$ denote the input sequence, temporal kernel, and bias. Conv1D denotes same-padded convolution along time, and ${g_{j}}^{l}$ sums the channel-wise outputs before the bias is added.

Channel aggregation preserves the ordered feature sequence.

Equation (14) applies ReLU without changing the sequence dimensions.

(14) $$x_{j}^{l}=ReLU\left(g_{j}^{l}\right)=max\left(0,g_{j}^{l}\right)$$

In Equation (14), $g_{j}^{l}$ is the pre-activation convolution output and $x_{j}^{l}$ is the rectified linear unit (ReLU) output, defined element-wise as $max\left(0,g_{j}^{l}\right)$.

Equation (15) summarizes the sequence-preserving interface between the convolutional encoder and LSTM.

(15) $$X\in R^{B\times 12\times 3}\to H_{CNN}\in R^{B\times 12\times F}$$

Here, *B* denotes batch size, 12 is the temporal length, and *F* is the number of convolutional feature channels selected by the joint validation search.

### 3.3. Sequence-Returning Long Short-Term Memory (LSTM)

Equations (16)–(23) define the standard LSTM update at each historical step.

(16) $$\sigma \left(x\right)=\frac{1}{1+e^{-x}}$$

(17) $$f_{t}=\sigma \left(W_{f}\left[h_{t-1},x_{t}\right]+b_{f}\right)$$

In Equations (16) and (17), σ is the logistic function, $x_{t}$ is the current input, $h_{t-1}$ is the previous hidden state, $W_{f}$ and $b_{f}$ are the forget-gate parameters, and $f_{t}$ is the gate vector. Matrix multiplication is used in the affine transform; element-wise products appear only in the state updates.

The input gate controls how much candidate information is written to the cell state. Equations (18)–(20) define the hyperbolic tangent, the input gate $i_{t}$, and the candidate cell state ${\tilde{c}}_{t}$.

(18) $$tanh\left(x\right)=\frac{e^{x}-e^{-x}}{e^{x}+e^{-x}}$$

(19) $$i_{t}=\sigma \left(W_{i}\left[h_{t-1},x_{t}\right]+b_{i}\right)$$

(20) $${\tilde{c}}_{t}=tanh\left(W_{c}\left[h_{t-1},x_{t}\right]+b_{c}\right)$$

The vectors $i_{t}$ and ${\tilde{c}}_{t}$ are computed from $\left[h_{t-1},x_{t}\right]$ using $W_{i}$, $b_{i}$, $W_{c}$, and $b_{c}$. The candidate state ${\tilde{c}}_{t}$ is distinct from the updated cell state $c_{t}$.

Equations (21)–(23) update the cell and output-gated hidden state.

(21) $$c_{t}=f_{t}⊙c_{t-1}+i_{t}⊙{\tilde{c}}_{t}$$

(22) $$o_{t}=\sigma \left(W_{o}\left[h_{t-1},x_{t}\right]+b_{o}\right)$$

(23) $$h_{t}=o_{t}⊙tanh\left(c_{t}\right)$$

Here, $c_{t}$ is the current cell state, $o_{t}$ is the output-gate vector, $h_{t}$ is the current hidden state, and $W_{o}$ and $b_{o}$ are the output-gate parameters; ⊙ denotes element-wise multiplication.

LSTM returns the complete historical hidden-state sequence to TSAN.

(24) $$H_{LSTM}=\left[h_{1},\ldots ,h_{T}\right]\in R^{B\times T\times U},T=12$$

In Equation (24), $h_{t}$ is the LSTM hidden state at historical step t, T=12, and U is the hidden-unit count. Changing the recurrent hidden width also changes the attention model dimension; this coupled-width comparison is controlled by U.

The recurrent hidden and cell states are initialized to zero for each input window. The proposed network retains the complete LSTM sequence for temporal attention. In the LSTM and CNN–LSTM baselines, the final hidden state feeds the direct three-output head; the temporal convolutional network (TCN) uses the representation at the final historical position. These output choices preserve the same direct three-location forecasting task.

### 3.4. Temporal Self-Attention Network (TSAN)

TSAN applies multi-head attention to the 12 historical LSTM states through query, key, and value projections. Its scores weight historical positions; they are neither spatial-location weights nor measures of causal importance.

(25) $$\{\begin{array}{c} Q=XW_{Q}\\ K=XW_{K}\\ V=XW_{V} \end{array}$$

In Equation (25), $X=H_{LSTM}\in R^{\left(B\times 12\times U\right)}$ is the LSTM output. The projection matrices $W_{Q},W_{K},W_{V},W_{O}\in R^{\left(U\times U\right)}$ produce full all-head projections $Q=XW_{Q}$, $K=XW_{K}$, and $V=XW_{V}$. Head-specific slices are formed from the corresponding column blocks.

(26) $$d_{k}=\frac{d_{model}}{h}$$

In Equation (26), $d_{model}=U$, the number of heads h=A, and $d_{k}=\frac{U}{A}$. Candidate decoding therefore retains only head counts that divide U; every evaluated pair U and A must satisfy this constraint.

(27) $$Attention\left(Q,K,V\right)=softmax\left(\frac{QK^{T}}{\sqrt{d_{k}}}\right)V$$

In Equation (27), $K^{T}$ is the transposed key matrix and $\sqrt{d_{k}}$ scales the dot products. Softmax is applied along the key-time axis for each query position.

(28) $${head}_{i}=Attention\left(XW_{Q}^{i},XW_{K}^{i},XW_{V}^{i}\right)$$

Equation (28) is equivalently ${head}_{i}=Attention\left(Q_{i},K_{i},V_{i}\right)$, where $Q_{i},K_{i},V_{i},{head}_{i}\in R^{\left(B\times 12\times d_{k}\right)}$ for i=1,…,A.

(29) $$MultiHead\left(X\right)=Concat\left({head}_{1},\ldots ,{head}_{A}\right)W_{O}$$

In Equation (29), the A heads are concatenated and projected by $W_{O}$. Residual normalization, a position-wise feed-forward layer, and a second residual normalization return a B×12×U sequence.

The query, key, value and output mappings are affine projections with learned biases; Equations (25) and (29) omit these additive biases for compactness. The parameter count includes all four bias vectors. Each attention block uses post-residual layer normalization. Its position-wise feed-forward mapping expands from *U* to 2*U* with ReLU and projects back to *U*, followed by a second residual addition and layer normalization.

Global average pooling reduces the TSAN sequence to one *U*-dimensional vector. A 64-unit rectified linear unit (ReLU) dense layer, dropout at 0.2, and a three-unit dense head produce the direct forecast for the selected dataset and horizon. Algorithm 1 specifies configuration selection and task-specific fitting.
**Algorithm 1:** Joint Six-Task Validation Search for a Shared Configuration and Direct Three-Location Prediction.Require: the Caltrans Performance Measurement System (PeMS) and Harbin datasets, each represented by a synchronized three-location speed matrix;Require: direct horizons H∈{1,3,6} and input length T=12;Require: ordered search vector P=[F,U,A] on F=16:8:128, U=32:8:256, A∈{1,2,4,8}; 1740 configurations in total;Input: N=10; $T_{max}=50$; k=0.1; b=0.3; $p_{roll}=0.9$; $p_{\alpha }=0.5$; β=1.5; S=0.5; candidate cap = 15 epochs; patience = 5;Seeds: candidate evaluation {17,29,43}; search {101,503,907,1301,1709}; final evaluation {42,2026,3407,7919,10007}.Output: one validation-selected common $P_{shared}$, six-task test predictions, and the search summaries;1: for each dataset D∈{PeMS,Harbin} do2:         Audit timestamps, location identifiers and continuous segments;3:         for each H∈{1,3,6} do; let q=(D,H);4:               Within each continuous segment, form $X_{t}\in R^{\left(12\times 3\right)}$ and $y_{t+H}\in R^{3}$;5:               Order nominal causal targets by target timestamp;6:               Freeze the first $\left\lfloor 0.70N_{nominal}\right\rfloor$ targets for training and the next $\left\lfloor 0.20N_{nominal}\right\rfloor$ for provisional validation; assign the remainder to testing, then apply the common three-location completeness mask within each fixed split; 6a:     end for; for the dataset, take the latest training-target time and earliest test-forecast origin across the three provisional horizon partitions; 6b:     for each horizon *H* in {1,3,6}, let *q* = (*D*,*H*); retain validation members only when their origin is at or after the common training cutoff and their target is at or before the common test-origin cutoff;7:               Fit the scaler using training-visible speeds and apply it unchanged to all splits;8:               for each grid configuration c and candidate seed $r_{c}\in \left\{17,29,43\right\}$ do9:                      Build Conv1D(F)–LSTM(U)–TSAN(A) with a direct three-location output head;10:                       Set candidate seed $r_{c}$; fit on training samples for at most 15 epochs and monitor validation RMSE with patience 5;11:                       Restore the best weights, inverse-transform validation outputs, and store the three-location macro RMSE $R_{c,q,r_{c}}$;12:               end for; for each configuration c, average its three candidate-seed macro RMSE values and divide by the same task’s Persistence validation macro RMSE to store $a_{c,q}$.13:         **end for**14: **end for**15: Freeze the common component matrix and define $J\left(c\right)=\frac{1}{6}\sum_{q\in Q}a_{c,q}$, where Q contains the six dataset–horizon tasks.16: for each search seed σ and method m (Random, PSO, DBO, IDBO, IDBO-w/o-Levy, or IDBO-w/o-Adaptive) do17:         Initialize the same N=10 proposals for all methods at search seed σ; for DBO-family methods retain the fixed 2/2/3/3 subgroups; at t=1, set $X_{i}\left(0\right)=X_{i}\left(1\right)$;18:         Decode [F,U,A] onto the stated grid by nearest indices (ties to even); A ∈ {1,2,4,8} divides U, and repair repeated configurations by the same deterministic map to an unused ID;19:         Look up the six stored components for the initial proposals; set $c_{0}$ to their minimum J, initialize $X^{W}$, $X^{*}$, $X^{l}$, $J_{min}$ and $P_{best}\left(m,\sigma \right)$, and set the non-improvement flag to false;20:         for iteration $t=1,\ldots ,T_{max}$ do; set $J_{prev}=J_{min}$;21:               **if** m is a DBO-family method **then**22:                      Update rolling with Equations (1)/(3) and their fixed rules; update breeding and foraging with Equations (5)/(7);23:                      For IDBO variants, compute ω(t) from Equation (12) once; set ω=1 for IDBO-w/o-Adaptive;24:                      **if** m is an IDBO variant and the preceding completed iteration did **not improve**
$J_{min}$
**then**25:                             Update stealing candidates using Equation (9), with Lévy factors from Equations (10) and (11); set Levy(s)=0 for IDBO-w/o-Levy;26:                      **else** update stealing candidates by the standard Equation (8); **end if**27:               **else** apply the specified PSO update, or for Random sample an unused configuration ID without replacement; **end if**28:               Decode and repair every candidate [F,U,A] as above; retain 10 unique initial proposals plus 50×10 unique subsequent proposals per method and search seed;29:               for each candidate $P_{i}$ do; look up its six normalized validation components and joint objective $J\left(P_{i}\right)$;30:                      if $J(P_{i})<J_{min}$, update $J_{min}$ and $P_{best}\left(m,\sigma \right)$; break exact joint-objective ties by configuration ID and retain the non-increasing best-so-far value $b_{p}$;31:               end for; refresh $X^{W}$, $X^{*}$, and $X^{l}$ for DBO-family methods; for IDBO variants set non-improvement = $J_{min}\geq J_{prev}$;32:         **end for**33: **end for**; retain all proposal-level components and $b_{p}$, and summarize $\frac{b_{510}}{c_{0}}$, $\frac{1}{510}\sum_{p=1}^{510}\frac{b_{p}}{c_{0}}$, and the five-seed sample standard deviation (SD) of $\frac{b_{510}}{c_{0}}$.34: Select $P_{shared}$ once from the union of configurations evaluated in the five IDBO search runs, minimizing (J(c),configurationID) lexicographically; fix it before final evaluation without using test outcomes;35: for each task q∈Q do36:         for each final seed r∈{42,2026,3407,7919,10007} do37:                   Rebuild the task-specific final network using $P_{shared}$, set r, and train for at most 30 epochs with validation early stopping;38:                   Restore the best weights; predict and inverse-transform the test split; store location-level RMSE, MAE, ε-MAPE, and signed ME;39:         **end for**40:         Report the five-seed arithmetic mean and sample SD, using equal-weight three-location macro metrics;41: **end for**42: Store the paired target-time predictions and observations for the statistical procedure in Section 4.3.3;43: **return** the common configuration $P_{shared}$, predictions for all six tasks, and the corresponding search summaries.

## 4. Experiments

The experiments use two independent synchronized three-location datasets: a PeMS freeway record and a Harbin urban-road record. Separate direct models predict 5, 15, and 30 min speeds within each dataset, giving six dataset–horizon tasks.

### 4.1. Datasets and Forecasting Protocol

#### 4.1.1. PeMS Three-Detector Corridor

The PeMS task uses three ordered freeway detectors, whose locations and model-channel labels are shown in Figure 4.

The three channels contain 10,080 synchronized 5 min speed observations. For PeMS, the forecasting tensor contains speed only; lane-1 flow and the derived flow-to-speed density ratio are retained for descriptive traffic-state analysis.

#### 4.1.2. Harbin Three-Location Urban-Road Case Study

The Harbin dataset was obtained from the Baidu Maps traffic-data interface, whose average-speed field reports road-segment mean speed in km/h at 5 min intervals in Asia/Shanghai local civil time. The ordered model channels are H1, Baojian Road from Haping Road to Xuefu Road (east to west); H2, Fudan Street from Xuefu Road to No. 4 Warehouse (east to west); and H3, Hashuang Road from Sanba Reservoir Bridge toward Haqian Road/Xinglong Road (east to south). Records were joined by exact nominal timestamps across the three segment identifiers. The channel order represents synchronized cross-location model context rather than a measured vehicle trajectory. The three Harbin Road segments and their ordered model context are shown in Figure 5.

The record contains 3036 nominal synchronized timestamps in 11 daily continuous segments from 10 May 2022 00:05 to 20 May 2022 23:00, with 276 five-minute timestamps per day. A common acquisition gap affects 19 timestamps from 20:20 to 21:50 on 19 May, corresponding to 57 road-level cells (0.626% of each series). For each road and missing clock time, imputation uses the median speed at the same clock time computed exclusively from available training-stage observations. The resulting road- and clock-time-specific statistic is fixed before validation and applied unchanged to missing historical inputs in validation and test partitions. Missing target values are excluded by a common three-location complete-target mask; imputed values are not evaluated as observed targets. The imputation statistic defined above remains fixed throughout evaluation; the common chronological split, training-fitted scaling, and validation-based model-selection procedures are summarized in Section 4.3.2 and Algorithm 1. Input windows and targets are formed within individual daily segments.

Table 1 summarizes data coverage, nominal sample counts, and the eligible task-specific partitions after target-completeness and common information-boundary filtering.

PeMS covers 35 days and Harbin covers 11 days. Across the three horizons, PeMS provides 10,063–10,068 nominal samples and 1007–1008 complete test targets; Harbin provides 2849–2904 nominal samples, 2825–2883 eligible samples, and 273–278 complete test targets. The validation boundary rule removes 34 of 7746 provisional validation members across the six tasks using timestamps alone. The resulting validation counts are 2007/2005/2002 for PeMS and 573/567/558 for Harbin at 5/15/30 min. Scaling uses training-visible observations only. These test partitions assess later observations at the same monitored locations; they do not constitute transfer tests to unobserved sites or seasons.

### 4.2. Data Characteristics and Cross-Location Dependence

Figure 6 characterizes PeMS speed variability and the descriptive flow–speed and density–speed relationships before forecasting.

On the selected 30 December high-variability block, the three detectors span 16.65–126.98 km/h; mean speeds are 85.79 km/h at P1, 94.99 km/h at P2, and 94.27 km/h at P3. The recurrent changes are broadly shared, but the levels and transition magnitudes remain location dependent.

Figure 7 summarizes the corresponding temporal and distributional characteristics of the Harbin dataset.

On the selected 20 May segment, mean speeds are 31.08, 34.08, and 35.81 km/h at H1, H2, and H3, respectively. The corresponding minimum speeds are 7.92, 6.16, and 19.31 km/h. Daily summaries are computed separately within each continuous daily segment using available observations.

To assess whether synchronized cross-location inputs are empirically supported, Figure 8 compares contemporaneous Pearson and Spearman associations with short-lag Pearson associations in both datasets.

PeMS shows off-diagonal Pearson correlations of 0.715–0.891 and Spearman correlations of 0.646–0.779. The P1 → P2 curve peaks at zero lag (r = 0.788). The P2 → P3 curve peaks at zero lag (r = 0.891). Harbin shows off-diagonal Pearson correlations of 0.497–0.622 and Spearman correlations of 0.620–0.677. The H1 → H2 curve peaks at +45 min (r = 0.575). The H2 → H3 curve peaks at −15 min (r = 0.567). A positive lag means that the first location leads the second. These lag patterns describe temporal associations rather than measured travel times or causal propagation; their forecasting value is assessed through the Self-only, Three-site, and Shuffled-context controls.

### 4.3. Experimental Design

#### 4.3.1. Comparison Models

Table 2 organizes a benchmark suite spanning persistence, vector autoregression (VAR), radial-basis-function support vector regression (RBF–SVR), long short-term memory (LSTM), a convolutional neural network–LSTM hybrid (CNN–LSTM), a temporal convolutional network (TCN), Transformer, its capacity-matched counterpart (Transformer-CM), and the proposed Improved Dung Beetle Optimizer-selected temporal self-attention network.

Persistence, VAR, and SVR provide deterministic reference results, whereas neural models are evaluated over five final seeds. Table 2 gives model-specific candidate domains. Baselines are selected separately for each dataset–horizon task by mean validation performance over the candidate seeds, with candidate ID breaking ties. Transformer-CM candidates first meet both parameter and MAC eligibility limits and then follow the same task-wise validation rule. Proposed alone uses one shared configuration selected with a six-task validation objective. Random Search, PSO, standard DBO, IDBO, and the two IDBO component controls share the search budget, while Section 4.4.1 compares forecasting models. All methods use the same target members, training-fitted per-channel scaling and evaluation rules; applicable neural models share the Adam training settings.

#### 4.3.2. Training, Search, Reporting, and Complexity

The reference implementation uses Python 3.11, TensorFlow 2.18, NumPy 2.0.2 and scikit-learn 1.8. Neural models use a direct three-location output with separately fitted parameters for each dataset and forecast horizon. The Adam learning rate, candidate and final epoch caps, stopping patience, dropout and seed groups are fixed in Table 3.

Table 3 consolidates the shared data, training, search, reporting and statistical settings. Parameter counts, analytical multiply–accumulate operations (MACs) per sample, and 32-bit floating-point (FP32) storage in mebibytes (MiB) describe the selected structures. Batch-one latency follows the disclosed analytical scenario. Paired inference uses the Diebold–Mariano test with the Harvey–Leybourne–Newbold correction and the horizon-specific heteroskedasticity- and autocorrelation-consistent lag.

The main forecasting comparisons use a 12-step input window. Chronological 70%/20%/remainder boundaries are fixed from nominal targets before target-completeness and the common cross-horizon validation boundary rule are applied. Per-channel z-score means and standard deviations are estimated from observed training speeds and applied unchanged to validation and test samples. Historical input imputation uses training observations only. Neural models minimize mean squared error (MSE) with the stated readout L2 penalty using Adam. Stochastic neural candidate evaluation uses three seeds and at most 15 epochs; final neural evaluation uses five separate seeds and at most 30 epochs, with patience five in both cases. The validation cache is constructed once from 1740 configurations, six tasks and three candidate seeds, corresponding to 31,320 nominal candidate training–validation evaluations and 10,440 seed-averaged task components. Each search method then accesses this fixed cache through 510 unique configuration queries per search seed, starting from the same ten initial proposals. The five search repetitions compare search access to a common objective; the cache construction is a separate offline cost. Every validation, stopping and selection step uses the final eligible validation members in Table 1. The joint objective averages the six validation macro RMSE ratios relative to Persistence evaluated on those same members. One shared configuration is selected from the union of five IDBO proposal sets by the lowest joint objective and then configuration ID: 64 convolutional filters, 128 recurrent–attention units, and four attention heads. The width comparison fixes the 64 filters and four heads and changes only the coupled recurrent–attention width between 64 and 128. The U128 reference is identical to Proposed in configuration, fitting conditions, and predictions.

The batch-one analytical proxy adds compute time at 100 million MAC/s to the time for transferring one copy of the FP32 parameter array at 100 MB/s, where MB denotes 1,000,000 bytes. The transfer volume is four bytes per parameter. Thus, the base time in milliseconds is 1000 times the sum of MACs divided by 100 million and four times the parameter count divided by 100 million. The same dimensionless cost factors 0.8, 0.9, 1.0, 1.1 and 1.2 multiply this base time for every model; they are prescribed scenarios rather than timing repetitions. Their median factor is 1.0 and their linearly interpolated 95th-percentile factor is 1.18. For Proposed, 240,387 parameters correspond to 961,548 transfer bytes, yielding a scenario median of 37.662 ms and p95 of 44.441 ms; the latter corresponds to 0.0148% of the 300,000 ms sampling interval under these assumptions. Table 3 gives the structure counts needed to apply the same calculation to every method. These scenario summaries describe the assumed computation and transfer costs. Acquisition, preprocessing, activation traffic and platform-specific launch overhead are excluded. Cache construction and search queries remain separate offline costs. Dropout at 0.2 is applied after the attention feed-forward block and Dense(64) layer in the proposed model and at the corresponding dense stages where applicable.

The final implementations are fixed before test evaluation. Baseline and Transformer-CM configurations follow their task-specific validation rules; for each method, the selected configuration is the same in all six tasks. VAR uses lag order and ridge coefficient: 6/0.04. SVR uses *C*, gamma and epsilon: 10.0/0.1/0.1. LSTM uses hidden units and readout regularization: 128/0.04. CNN-LSTM uses convolutional filters, recurrent units and readout regularization: 64/128/0.04. TCN uses channels and readout regularization: 48/0.04. Transformer uses model width, feed-forward width, heads, encoder depth and readout regularization: 64/128/4/2/0.04. Transformer-CM uses model width, feed-forward width, heads, encoder depth and readout regularization: 144/104/4/2/0.04. The proposed model uses 64 same-padded kernel-3 filters, 128 recurrent units, four temporal-attention heads, attention model width 128 and feed-forward width 256, and a 64-unit dense layer in all six tasks. ReLU is used in convolutional and feed-forward blocks, with dropout 0.2 at the applicable attention/feed-forward and dense stages.

SVR flattens each 12-step, three-location history into 36 input features and uses one RBF regressor per output location. For analytical complexity, each regressor is assigned 512 support slots. With three outputs, a slot stores 36 feature values and one coefficient, with one additional intercept per output. Let *S* denote the 512 support slots per output and *D* the 36 input features. The parameter-equivalent storage count and operation proxy are defined below. These slots specify the cost proxy; they are not measured support-vector counts from fitted regressors.

$$P_{SVR}=3[S(D+1)+1]=56,835$$

$$M_{SVR}=3S(2D+2)=113,664$$

Both Transformer variants linearly project each three-location input vector to the selected model width and add fixed sinusoidal positional encodings for the historical positions. Two encoder layers preserve the sequence length. Global average pooling over the historical positions yields one model-width vector, and a linear head maps it directly to three simultaneous location forecasts. Output columns follow P1, P2, P3 for PeMS and H1, H2, H3 for Harbin. Each horizon has a separate model. The fixed positional encoding and pooling introduce no trainable parameters.

The fixed positional code is defined in Equations (30) and (31) for historical position *t* from 0 to 11 and channel-pair index *j* from 0 to *d*/2−1. The widths *d* are 64 for Transformer and 144 for Transformer-CM. Positional additions and equal-time averaging introduce no learned parameters and are excluded from the stated MAC convention.

(30) $$PE(t,\;2j)=sin(\frac{t}{{10,000}^{\frac{2j}{d}}})$$

(31) $$PE(t,\;2j+1)=cos(\frac{t}{{10,000}^{\frac{2j}{d}}})$$

Readout L2 regularization applies only to the final linear three-output weight matrix. In Equation (32), *N* is the number of fitting examples; *y* and *ŷ* are the location-specific standardized target and prediction, indexed by example *i* and location *c*; and *w* with subscript *c* is column *c* of the output weight matrix. Both terms use equal-output normalization, and *λ* is 0.04. Output biases and all preceding convolutional, recurrent, attention, feed-forward and intermediate Dense(64) weights are excluded. The output matrix has 64 rows for Proposed and the model width for each Transformer variant. Predictions are inverse-transformed before validation scoring and error reporting. The coefficient is distinct from optimizer-wide weight decay and VAR ridge regularization.

(32) $$L=\frac{1}{3N}\sum_{i=1}^{N}\sum_{c=1}^{3}{\left({ŷ}_{i,c}-y_{i,c}\right)}^{2}+\frac{\lambda }{3}\sum_{c=1}^{3}{\|w_{c}{\|}_{2}}^{2}$$

PSO operates in the normalized three-coordinate search space with zero initial velocities. Its inertia weight is 0.96 minus 0.08 times the completed-iteration fraction; the cognitive and social coefficients are 0.18 and 0.22. Each coordinate uses independent uniform random factors between zero and one. Updated positions are clipped to the unit cube and decoded to the nearest grid level, with exact half-grid ties resolved to the even index. Velocities are retained without a separate clamp, and duplicate configurations use the common deterministic replacement rule.

The capacity-matched comparison assesses whether the predictive advantage persists at a similar parameter and operation budget. Transformer-CM structures must satisfy parameter and MAC ratios each within [0.92, 1.08] relative to the shared Proposed configuration before validation selection; coverage requires at least eight eligible structures for every reference in the 1740-point grid. Ten initial proposals plus 50 iterations of ten proposals are common to all six search methods. Across six methods and five search seeds, the comparison comprises 15,300 configuration queries and 91,800 task-component accesses to the shared cache. These counts exclude its one-time construction and do not measure an end-to-end reduction in model-training time. Section 4.4.1 compares the nine forecasting implementations under the common evaluation protocol.

#### 4.3.3. Metrics and Statistical Inference

Mean absolute error (MAE), root mean square error (RMSE), denominator-bounded mean absolute percentage error (ε-MAPE), and signed mean error (ME) are computed for each location. Residuals are defined as prediction minus observation, so positive ME indicates overprediction. The denominator bound in ε-MAPE prevents near-zero observed speeds from dominating the percentage error. A three-location macro value is the arithmetic mean of the three location-level metrics, giving each location equal weight. Five runs quantify initialization variability and are not treated as five independent observations for a *t*-test. Transformer and Transformer-CM are specified as separate comparators before test evaluation. At each target timestamp, paired squared-error differences are averaged over seeds and locations to form one task-level loss-difference series. The resulting loss-difference series is evaluated using the Diebold–Mariano (DM) test [21]. The Harvey–Leybourne–Newbold (HLN) correction is then applied to the DM statistic [22]. A heteroskedasticity- and autocorrelation-consistent (HAC) lag equal to H−1, where *H* is the forecast horizon in five-minute steps, is used, with Student-t inference using *N* − 1 degrees of freedom. The 5, 15 and 30 min horizons therefore use lags 0, 2 and 5. Bartlett weights are applied to autocovariances paired by exact five-minute target-index gaps within continuous segments. *N* is the number of paired target timestamps; locations and runs are averaged within each timestamp. Holm correction is applied separately within each comparator’s six dataset–horizon comparisons [23]. A moving-block bootstrap is used to obtain the 95% confidence interval for relative RMSE improvement [24]. Resampling is performed on the target-time series within continuous segments in blocks of 12. For each of 5000 resamples, location-level RMSE is computed separately for each paired run and then averaged equally over the three locations and five runs for each method; the relative difference between the two method means, using the comparator mean as the denominator, is the bootstrap statistic. The two-sided 95% intervals use the bootstrap percentiles. These intervals are not multiplicity-adjusted. The bootstrap quantifies RMSE-gain uncertainty separately from the horizon-based HAC specification; its resample count does not assess sensitivity to alternative HAC lags.

(33) $$e_{t,s,j}^{\left(m\right)}={\hat{y}}_{t,s,j}^{\left(m\right)}-y_{t,j}$$

(34) $${\mathrm{MAE}}_{s,j}^{\left(m\right)}=\frac{1}{N}\sum_{t=1}^{N}\left|e_{t,s,j}^{\left(m\right)}\right|$$

(35) $${\mathrm{RMSE}}_{s,j}^{\left(m\right)}=\sqrt{\frac{1}{N}\sum_{t=1}^{N}{\left(e_{t,s,j}^{\left(m\right)}\right)}^{2}}$$

(36) $${\varepsilon \text{-}\mathrm{MAPE}}_{s,j}^{\left(m\right)}=\frac{100}{N}\sum_{t=1}^{N}\frac{\left|e_{t,s,j}^{\left(m\right)}\right|}{\max\left(\left|y_{t,j}\right|,\varepsilon \right)}$$

(37) $${\mathrm{ME}}_{s,j}^{\left(m\right)}=\frac{1}{N}\sum_{t=1}^{N}e_{t,s,j}^{\left(m\right)}$$

(38) $${\bar{M}}^{\left(m\right)}=\frac{1}{S}\sum_{s=1}^{S}\left(\frac{1}{3}\sum_{j=1}^{3}M_{s,j}^{\left(m\right)}\right)$$

(39) $$L_{t}=\frac{1}{3S}\sum_{s=1}^{S}\sum_{j=1}^{3}\left[{\left(e_{t,s,j}^{\left(C\right)}\right)}^{2}-{\left(e_{t,s,j}^{\left(P\right)}\right)}^{2}\right]$$

In Equations (33)–(39), *t* indexes the *N* target timestamps, *s* indexes the *S* runs, and *j* indexes the three locations. The prediction residual is prediction minus observation; *ε* is 5 km/h. *M* denotes any of the four location-level metrics, so each run is first macro-averaged over the three locations and the reported mean is then computed across runs. *S* is five for stochastic models and one for deterministic baselines; sample standard deviations are reported only for the five-run results. For the paired loss difference, *C* denotes the comparator and *P* the proposed method, with the same timestamps, locations, and paired runs. Positive $L_{t}$ means lower squared loss for *P* at timestamp *t* after averaging the paired location-run series. ε-MAPE is expressed in percent; MAE, RMSE, and signed ME are in km/h.

### 4.4. Comparative Results

#### 4.4.1. Multi-Horizon Main Benchmark

Using the disclosed protocol, Table 4 compares all nine methods by equal-weight three-location macro RMSE across the six dataset–horizon tasks.

The proposed configuration yields the lowest three-location macro RMSE among the nine benchmark methods in all six tasks. Its five-run mean ± sample SD values at 5, 15, and 30 min are 3.705 ± 0.026, 4.758 ± 0.049, 5.752 ± 0.070 km/h on PeMS and 1.694 ± 0.024, 2.323 ± 0.030, 2.736 ± 0.032 km/h on Harbin. Relative to the validation-selected Transformer, macro RMSE reductions are 13.97–14.42% on PeMS and 12.79–13.08% on Harbin. Relative to the validation-selected Transformer-CM, the reductions are 7.55–7.92% and 6.66–7.24%, respectively. Each reduction uses the corresponding comparator RMSE as the denominator. A local reversal remains for Transformer-CM at Harbin-15, location H3, seed 7919: its RMSE is 2.027 km/h versus 2.046 km/h for Proposed. The six task-level mean orderings remain unchanged.

Figure 9 gives the complete MAE, epsilon-MAPE and signed ME results for the same nine methods and six tasks, with task columns grouped into PeMS and Harbin. Each stochastic result has an equal-location macro mean and the sample SD over five runs; Persistence, VAR and SVR each have one deterministic value. The SDs describe run-to-run variation. Lower MAE and epsilon-MAPE indicate lower error, while ME closer to zero indicates smaller systematic bias. Negative and positive ME denote underprediction and overprediction. Color is normalized separately within each metric–task column over the nine methods, using error magnitude and |ME| for signed bias. Bold means identify the full-precision minimum; the emphasized Proposed label identifies the focal method.

The proposed method has the lowest three-location macro MAE and epsilon-MAPE in all six tasks. Its signed macro ME ranges from −0.051 to 0.008 km/h. The mean absolute value of these six signed task means is 0.014 km/h. In absolute ME, Proposed ranks first among the nine methods in PeMS at 5 min and in all three Harbin tasks, and second in PeMS at 15 and 30 min, where Persistence has the smaller absolute bias. Absolute ME measures systematic bias and can be small when positive and negative errors cancel; its ordering is distinct from RMSE, MAE and epsilon-MAPE. For each method, the six-task bias summary first takes the absolute value of each signed task mean and then averages the six absolute values. Taking the absolute value after averaging signed task errors would allow further cancelation.

To examine horizon sensitivity beyond the tabulated rankings, Figure 10 plots the macro RMSE of all nine forecasting methods and its percentage change relative to the 5 min horizon.

Mean RMSE increases with horizon in all 18 dataset–method series. At 15 and 30 min, the proposed method’s mean within-run RMSE increases relative to the same run at 5 min are 28.42% and 55.25% on PeMS and 37.13% and 61.52% on Harbin, respectively. These percentages are averaged over the five matched seeds. The proposed model retains the lowest macro RMSE among the nine benchmark methods in all six tasks. Its five-run sample SD ranges from 0.024 to 0.070 km/h and describes variation across the recorded runs under the fixed evaluation protocol.

#### 4.4.2. Representative Prediction Trajectories

To complement aggregate errors with time-resolved behavior, Figure 11 shows 4 h test windows chosen on the first chronologically eligible date at fixed start times for each location. Eligibility requires 49 continuous target timestamps shared across the three horizons.

RMSE is computed after averaging the five prediction curves at each displayed timestamp. The resulting curve RMSE values are PeMS at 5 min: 1.258, 1.364, 2.274; PeMS at 15 min: 2.176, 1.797, 2.572; PeMS at 30 min: 1.993, 2.598, 3.905; Harbin at 5 min: 1.167, 1.314, 1.721; Harbin at 15 min: 1.545, 1.929, 2.502; Harbin at 30 min: 2.290, 1.996, 3.089 km/h, in the order Proposed, Transformer and Transformer-CM. Proposed has the lowest curve RMSE among these three methods in 4 of the six panels. These fixed-clock windows complement the complete test-set results in Table 4. The RMSE of an averaged curve is distinct from the mean of five individual-run RMSE values; the selected curves do not establish accurate tracking of every abrupt change by every run.

#### 4.4.3. Statistical Significance

To assess whether task-level gains persist after accounting for temporal dependence and multiple comparisons, Table 5 reports adjusted loss-differential tests and block-bootstrap effect estimates.

After separate six-task Holm adjustments, all 12 task-level comparisons meet the 0.05 criterion using unrounded *p*-values. The Harbin-30 comparison with Transformer-CM is just below the boundary and rounds to 0.050; it should be interpreted as borderline. Across the two comparator families, paired RMSE gains range from 6.66 to 13.08% in Harbin and 7.55 to 14.42% in PeMS. All 12 unadjusted bootstrap intervals have positive lower bounds. Significance is determined from the Holm-adjusted DM–HLN *p*-values, whereas the unadjusted bootstrap intervals quantify uncertainty in the RMSE-gain estimates. Local reversals remain: 48 of 84 descriptive PeMS-30 blocks have a negative mean paired squared-loss difference against Transformer-CM. This count is separate from the inferential target-time sample size and the bootstrap-resample count.

#### 4.4.4. Matched-Budget Optimizer Convergence

To examine search behavior under a common finite query budget, Figure 12 compares the recorded best-so-far joint validation histories of six search methods across five search seeds using the fixed validation cache defined in Section 4.3.2. Random search provides a non-adaptive reference for configuration selection [25]. The joint objective averages six task-level validation RMSE ratios relative to Persistence. Each seed shares the same ten initial configurations across methods. For the summary statistics, each history is divided by its best objective after these ten initialization evaluations, denoted *c*_10_. The normalized endpoint, AUC (the mean normalized best-so-far value over all 510 queries), and sample SD of normalized endpoints describe final quality, search progress, and run-to-run variation. All query positions are retained as steps without smoothing or interpolation.

Across five search seeds, IDBO has the lowest mean normalized endpoint (0.829636) and AUC (0.869405). Relative to Random, PSO and DBO, its endpoint reductions are 9.63%, 7.38% and 8.82%; the corresponding AUC reductions are 7.16%, 4.74% and 6.39%, using each comparator mean as the denominator. Against the controls without Lévy perturbation and without adaptive weighting, the endpoint reductions are 3.69% and 8.52%, and the AUC reductions are 4.43% and 6.38%, respectively. Across the five comparator means, endpoint reductions range from 3.69% to 9.63% and AUC reductions from 4.43% to 7.16%. The selected F64/U128/A4 objective is 0.571285, equal to the full-grid minimum. The complete histories retain plateaus and intermediate reversals: seed 1301 trails a comparator at 113 of the 510 query positions. IDBO has the second-smallest sample SD of normalized endpoints (0.000708) among the six methods. All five IDBO runs reach the same cached optimum, giving zero SD for their raw endpoint objectives; the nonzero normalized SD arises from their different initial best values *c*_10_. PSO ends at two nearly equal cached scores, with raw endpoint SD 0.00000173. Exact two-sided paired sign-flip tests give *p* = 0.0625 for both endpoint and AUC against each of the five comparators. These five-seed optimizer comparisons are therefore not described as statistically significant at 0.05.

### 4.5. Controlled Analyses

#### 4.5.1. Out-of-Sample Calibration and Residual Diagnostics

Figure 13 examines value calibration and residual behavior for Transformer, Transformer-CM, and the proposed direct three-location model. Predictions are first averaged over the five runs at each target timestamp and location. For each method and dataset–horizon task, ten bins are formed from ranked held-out mean predictions using the fixed rank rule. Each bin compares the mean prediction and observation of its own paired members; bin membership can differ between methods. Positive prediction-minus-observation gaps denote overprediction. Residual medians and Q1–Q3 intervals summarize run-averaged predictions, with the interval covering the middle 50% of residuals; the five individual macro signed ME values are shown separately. Residual spread and run-level bias use separate scales, each shared across datasets. These held-out diagnostics do not enter model fitting or selection.

For Proposed, the mean absolute calibration gap over ten bins ranges from 0.115 to 0.478 km/h across the six tasks. Five-run mean macro signed errors range from −0.051 to 0.008 km/h. The population SD of run-averaged residuals is 2.528, 3.164, 3.821 km/h on PeMS and 1.154, 1.587, 1.801 km/h on Harbin at 5, 15 and 30 min, respectively. These SDs pool target timestamps and locations after averaging the five prediction runs. They differ from the five-run sample SD of macro errors and from the residual interquartile ranges in Figure 13, which span 1.040–3.508 km/h. Each method uses its own prediction-rank bins. A narrower aggregate residual distribution does not establish lower far-tail error in every individual run.

#### 4.5.2. Location-Level Cross-Site Context Effects

For the context comparisons, each target location retains the same backbone dimensions, history length and seed. Three-site uses synchronized histories from all three locations. Self-only retains the target history while suppressing information from the other two locations, with input dimensions and the target output held fixed. Shuffled-context retains the target history and divides each visible non-target history into three-step blocks, preserving order within each block. Both non-target locations share the same block permutation. For the 12-step input, four blocks are permuted; 6- and 24-step controls use two and eight blocks. No sample, continuous-segment boundary or forecast origin is crossed. Seed 73,021 and stable dataset–sample–target keys fix the permutation before fitting and prediction.

Three-site input reduces mean RMSE in all 18 location–horizon comparisons, with gains of 1.56–19.57% relative to Self-only. Shuffled-context gives 14 negative and 4 positive gains, ranging from −8.52% to 1.08%. Figure 14 retains paired-run variation. At the three-location macro level, Three-site has the lowest RMSE, MAE and epsilon-MAPE in all six tasks. Signed bias behaves differently: Three-site ranks first in five tasks and third at Harbin-5. Shuffled-context improves on Self-only in Harbin-15 epsilon-MAPE among the 18 macro error-magnitude comparisons. These results support the predictive value of synchronized context within the monitored locations.

#### 4.5.3. Structural and Coupled-Width Comparisons

To examine structural choices, Figure 15 compares the full model with separate removal of its convolutional, recurrent, or temporal-attention component across all six dataset–horizon tasks. Removing CNN passes the 12-step, three-channel input directly to the 128-unit LSTM and retains the attention and readout stages. Removing the recurrent layer retains the 64-filter CNN and applies a time-shared learned affine projection from 64 to 128 channels independently at each historical step; the resulting 12-step, 128-channel sequence enters the unchanged attention block. Removing temporal attention also removes its feed-forward and normalization stages; the 12 LSTM states are averaged over time and passed to the retained 64-unit dense layer and three-output head. This differs from the separately tuned CNN–LSTM baseline, whose final hidden state feeds a direct linear three-output head. The separate width comparison fixes 64 convolutional filters and four attention heads and changes the coupled recurrent–attention width between 64 and 128. The U128 reference is the full proposed configuration. IDBO component-removal controls are evaluated separately in Figure 12.

Across the six tasks, separate removal of CNN, the recurrent layer and temporal attention increases three-location macro RMSE, MAE and epsilon-MAPE in every task. Their equal-task mean relative RMSE increases are 12.96%, 16.94% and 10.49%, respectively. The full model has the smallest absolute five-run mean signed ME in five tasks; attention removal is closer to zero at Harbin-5. Some attention-removal runs also have lower MAE or epsilon-MAPE at PeMS P3. In the separate width comparison, U64 increases macro RMSE by 12.95–34.45% across tasks, with an equal-task mean of 21.21%, using each U128 task RMSE as the denominator. The width and capacity-matched comparisons use different reference configurations; Figure 12 separately evaluates IDBO under the common candidate evaluator and query budget.

#### 4.5.4. Window Sensitivity, Speed States, and Input Noise

Figure 16 evaluates 6-, 12-, and 24-step input windows, location-specific training-target speed tertiles fixed for testing, and zero-mean Gaussian input noise generated from common seeded innovations at 0, 0.05, and 0.10 times the training input-scaler standard deviation.

Among the 6-, 12- and 24-step histories evaluated on common eligible members, the 12-step input has the lowest macro RMSE, MAE and epsilon-MAPE in all six tasks. Relative to that reference, the 6-step input increases RMSE by 8.81–11.67% and the 24-step input by 4.49–6.99%. The 12-step input also has the smallest absolute five-run mean signed ME in five tasks. At 5 min, state-specific RMSE values are PeMS: low 4.085, medium 3.026, high 3.625 km/h; Harbin: low 1.776, medium 1.587, high 1.594 km/h. Noise levels of 0.05 and 0.10 times the training input-scaler standard deviation increase RMSE by 6.05% and 19.00% on PeMS, and 8.29% and 18.27% on Harbin, using each zero-noise RMSE as the denominator. Zero noise has the lowest macro RMSE, MAE and epsilon-MAPE in both five-minute tasks. Zero noise also has the smallest absolute mean signed ME in both tasks. These findings support the common 12-step specification within the tested range; signed bias and individual seed–location comparisons need not follow every aggregate error ordering.

### 4.6. Discussion

The comparative and controlled results support the integrated local–recurrent–attention configuration. Convolution represents short-range variation, recurrent processing preserves sequence order, and temporal attention weights the historical states. The structural and context comparisons are consistent with these complementary roles under the common protocol.

The independently trained freeway and urban-road tasks assess performance consistency within two monitored cases. The paired comparisons and synchronized-context results support the reported improvements under later-time evaluation at the same locations. The short records and three-location inputs define the scope of that evidence; transfer to unseen locations, new cities and longer observation periods requires further evaluation.

The accuracy gain is accompanied by a capacity cost: Proposed contains 240,387 parameters and 2,804,672 analytical MACs per sample, approximately 3.57 and 3.40 times the Transformer values. Transformer-CM has 229,603 parameters and 2,798,064 MACs, respectively 4.49% and 0.24% below Proposed and within both matching intervals. Its higher mean RMSE shows that matching the two complexity measures does not remove the observed forecasting difference. The substantial U64–U128 degradation separately shows the importance of the coupled recurrent–attention width under fixed F64/A4 conditions. These two controlled contrasts support a remaining architecture-level advantage while identifying width as an important factor. The optimizer is assessed through the common-evaluator search comparison in Section 4.4.4.

Configuration search and cache construction are performed offline; subsequent forecasts use only the selected network. Parameter and analytical MAC counts characterize the selected network’s computational scale. The scenario p95 is 44.441 ms relative to a 300,000 ms sampling interval under the stated compute and transfer assumptions (Section 4.3.2). End-to-end timing additionally depends on acquisition, preprocessing, activation traffic and platform overhead, which this proxy does not quantify. Figure 10 and Figure 16 characterize horizon, speed-state, history-length and input-noise sensitivity. The case studies use relatively short, speed-only records; Section 4.1.2 details the training-derived imputation protocol for Harbin.

## 5. Conclusions

This study presents an Improved Dung Beetle Optimizer-selected convolutional–recurrent temporal-attention framework for separate direct traffic-speed models at 5, 15, and 30 min horizons. Six monitored locations are organized as two independent synchronized three-location case studies: a PeMS freeway dataset and a Harbin urban-road dataset. The framework is evaluated under a chronologically isolated, validation-driven protocol across six separately trained dataset–horizon tasks.

Proposed has the lowest equal-location macro RMSE, MAE and epsilon-MAPE among the nine forecasting methods in all six tasks. Its RMSE reductions relative to Transformer are 13.97–14.42% on PeMS and 12.79–13.08% on Harbin; all 12 comparisons meet the 0.05 criterion using unrounded values after their separate six-task Holm adjustments; the Harbin-30 comparison with Transformer-CM lies just below the boundary. The structural, matched-capacity, coupled-width and synchronized-context comparisons support the integrated configuration and its selected settings. IDBO yields the lowest mean endpoint and AUC under the common query budget, while the reported seed-level exceptions distinguish aggregate advantage from uniform dominance. Together, these findings demonstrate consistent forecasting gains in the evaluated freeway and urban-road cases.

The accuracy gain entails a clear complexity trade-off, as the proposed network has greater parameter and analytical MAC counts than Transformer. Errors increase with forecast horizon and are largest in the low-speed groups of the five-minute state comparisons. Input perturbations and history length affect the error magnitude and signed bias differently, as the reported local exceptions show. Future work will extend temporal and spatial coverage, incorporate exogenous variables, and evaluate alternative training-derived imputation and drift protocols.

## Figures and Tables

**Figure 3:**
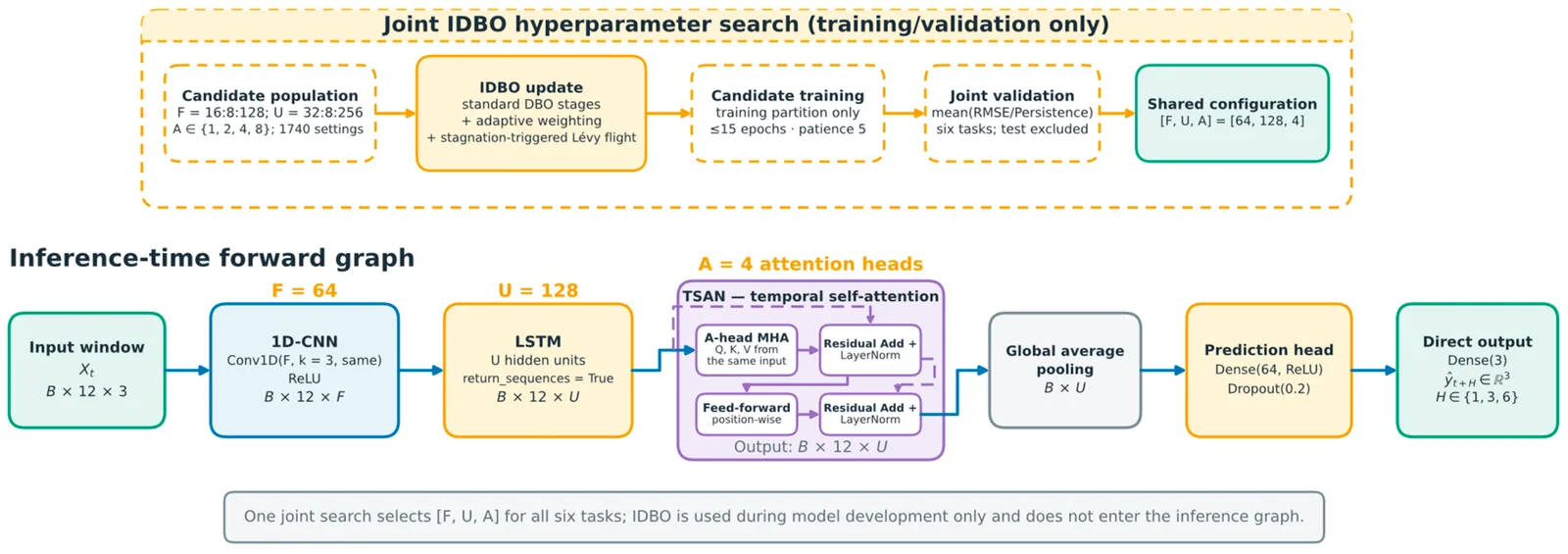
Offline configuration selection and the convolutional–recurrent temporal-attention architecture for a direct three-location forecast from a 12-step history. MHA and ReLU denote multi-head attention and rectified linear unit, respectively. Solid arrows indicate functional dependencies in the upper panel and forward information flow in the lower panel; dashed purple arrows denote residual connections within TSAN.

**Figure 4:**
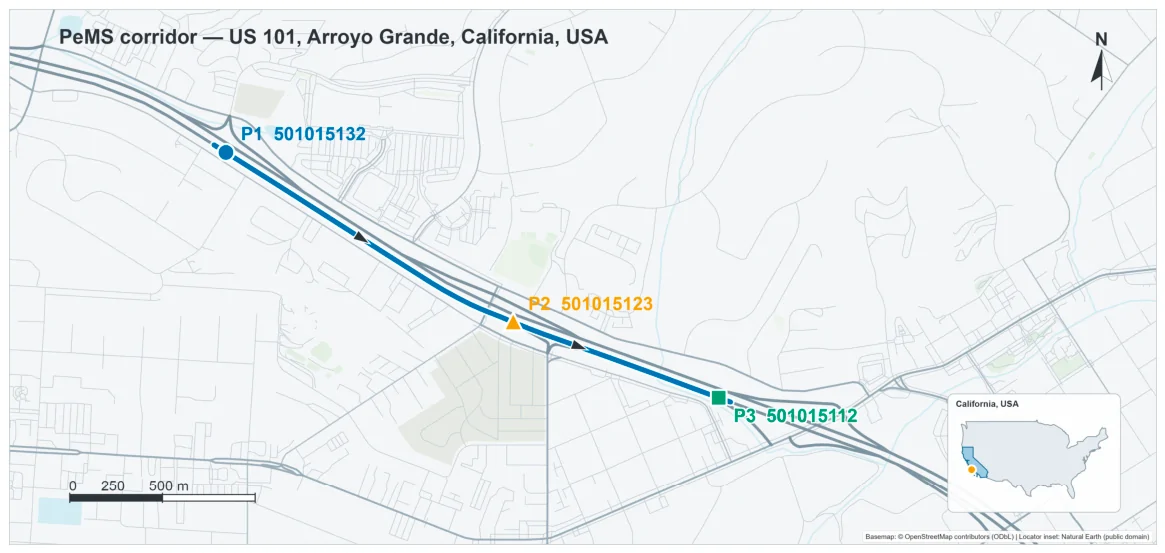
Ordered Caltrans Performance Measurement System (PeMS) detector corridor: P1 = 501015132, P2 = 501015123, and P3 = 501015112. Arrows indicate corridor order. Basemap © OpenStreetMap contributors under the Open Database License (ODbL); locator inset from Natural Earth (public domain). Detector markers indicate approximate positions reconstructed from the supplied locator map.

**Figure 5:**
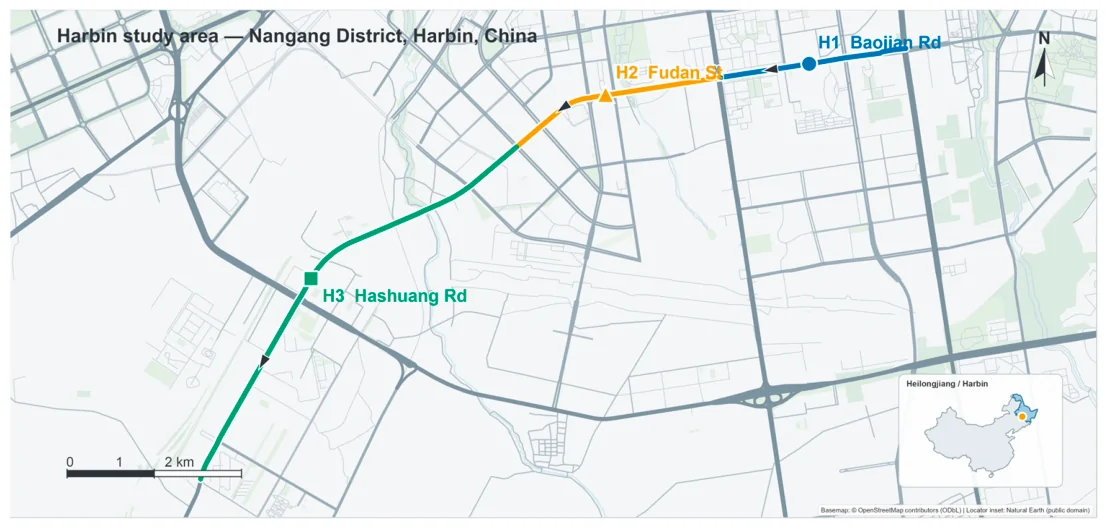
Harbin study locations in model-channel order: H1, Baojian Road; H2, Fudan Street; and H3, Hashuang Road. Basemap © OpenStreetMap contributors under the Open Database License (ODbL); locator inset from Natural Earth (public domain). Colored traces denote separate monitored road segments, and arrows indicate within-segment reporting directions; channel order does not imply a continuous vehicle trajectory.

**Figure 6:**
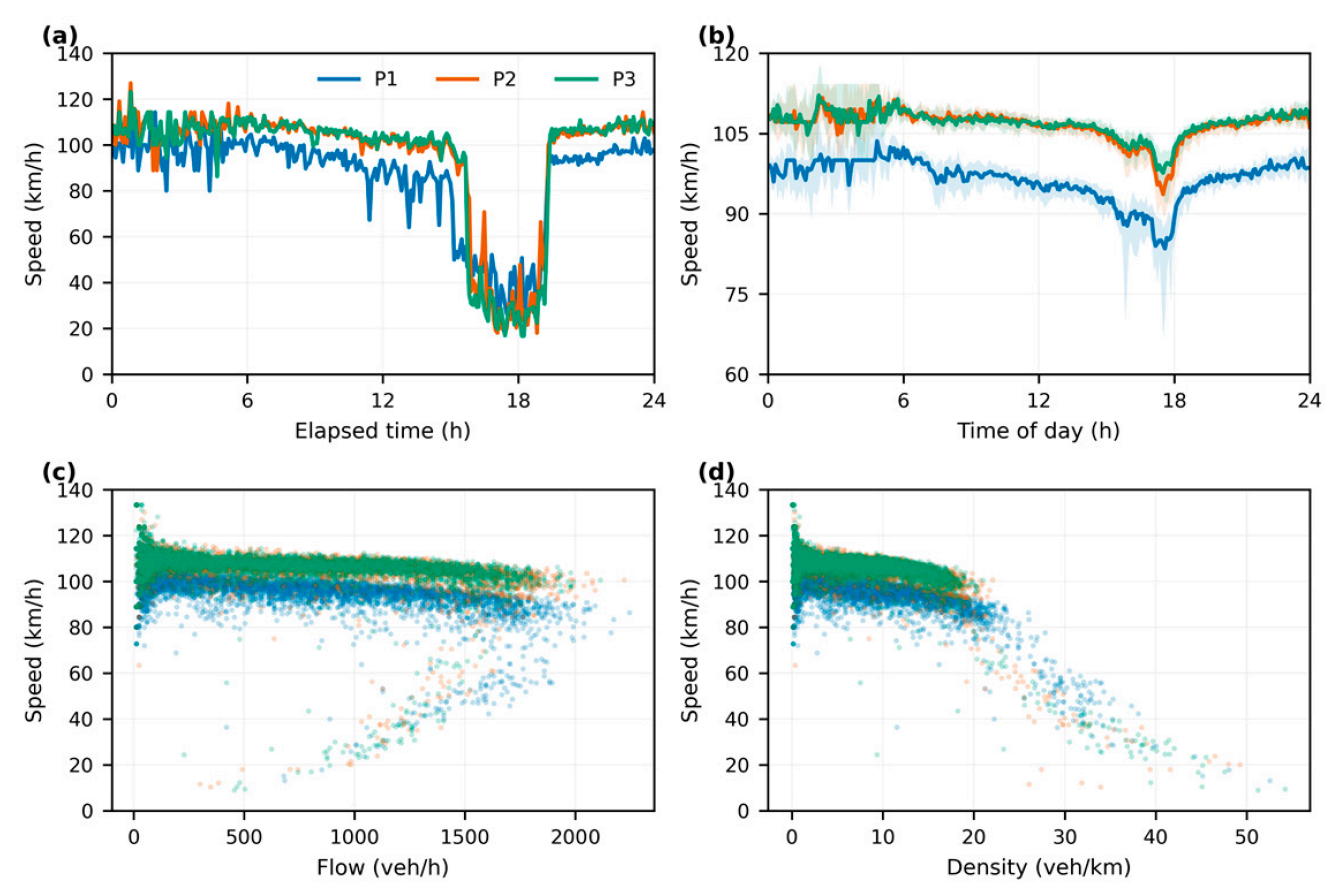
Caltrans Performance Measurement System (PeMS) data characteristics: (**a**) representative three-detector speed trajectories; (**b**) median intraday speed with interquartile bands; (**c**) flow–speed relationships; and (**d**) density–speed relationships.

**Figure 7:**
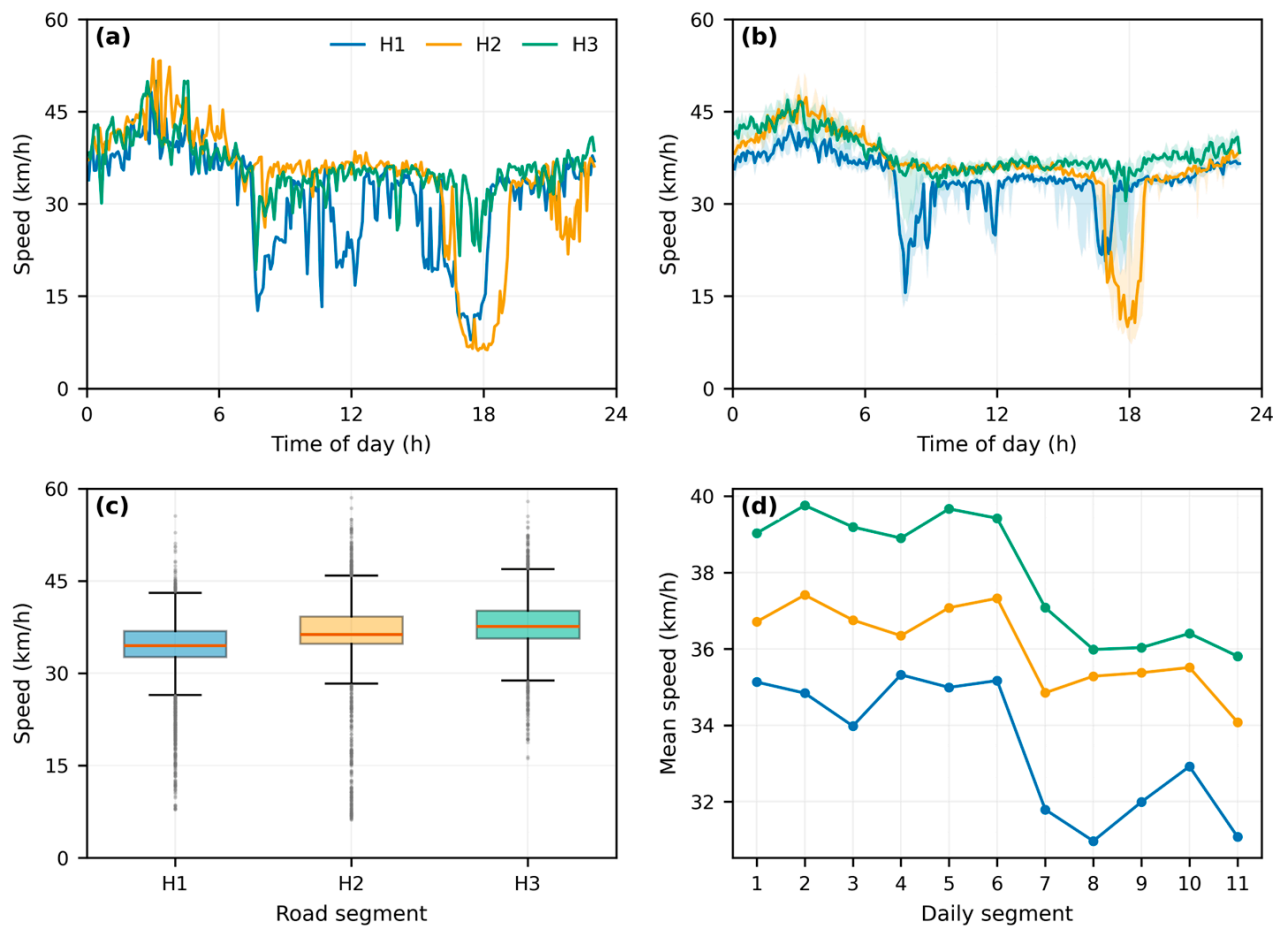
Harbin data characteristics: (**a**) representative three-road speed trajectories; (**b**) median intraday speed with interquartile bands; (**c**) speed distributions; and (**d**) daily mean speeds. Boxplot whiskers use 1.5 times the interquartile range (IQR), with outliers shown individually. In (c), the orange horizontal lines within the boxes indicate the medians.

**Figure 8:**
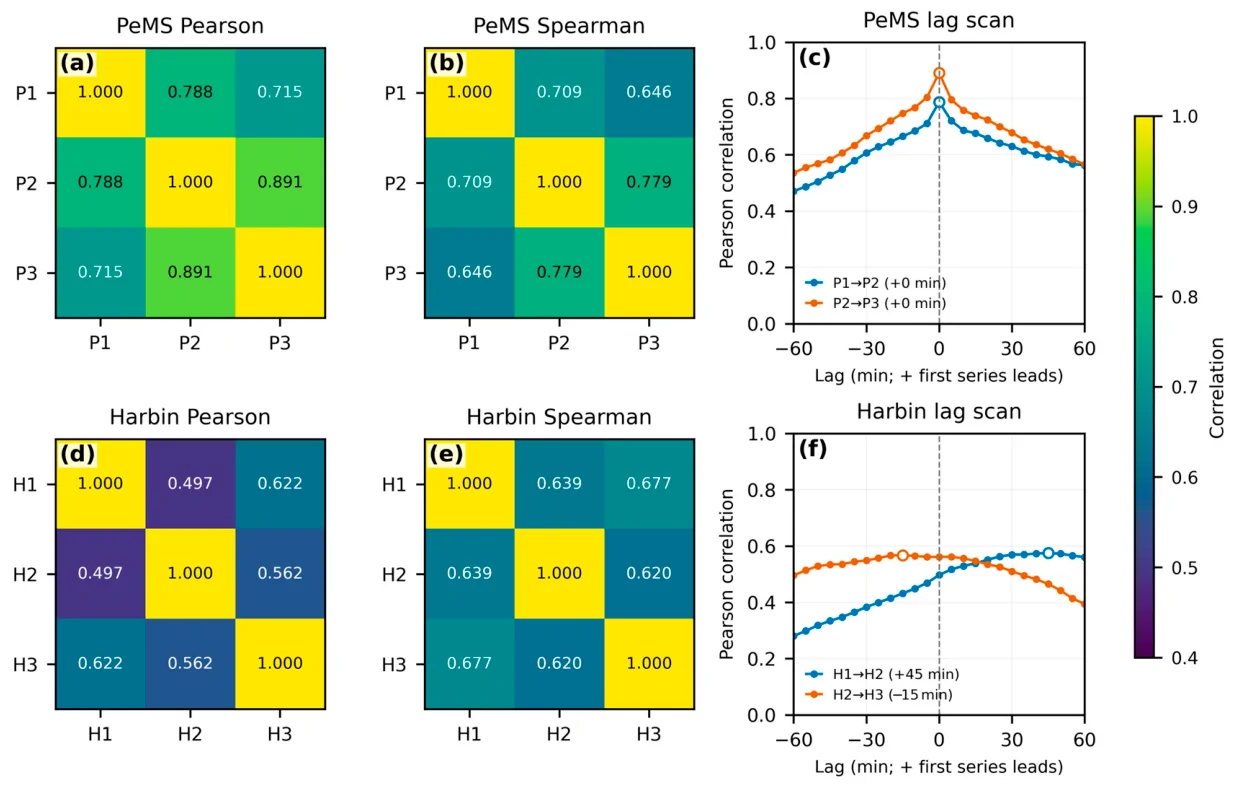
Cross-site speed dependence in the freeway (**a**–**c**) and urban-road (**d**–**f**) datasets: Pearson matrices (**a**,**d**), Spearman matrices (**b**,**e**), and lagged Pearson correlations for successive ordered location pairs over −12 to +12 five-minute steps (**c**,**f**). Positive lag indicates that the first-listed series leads the second-listed series.

**Figure 9:**
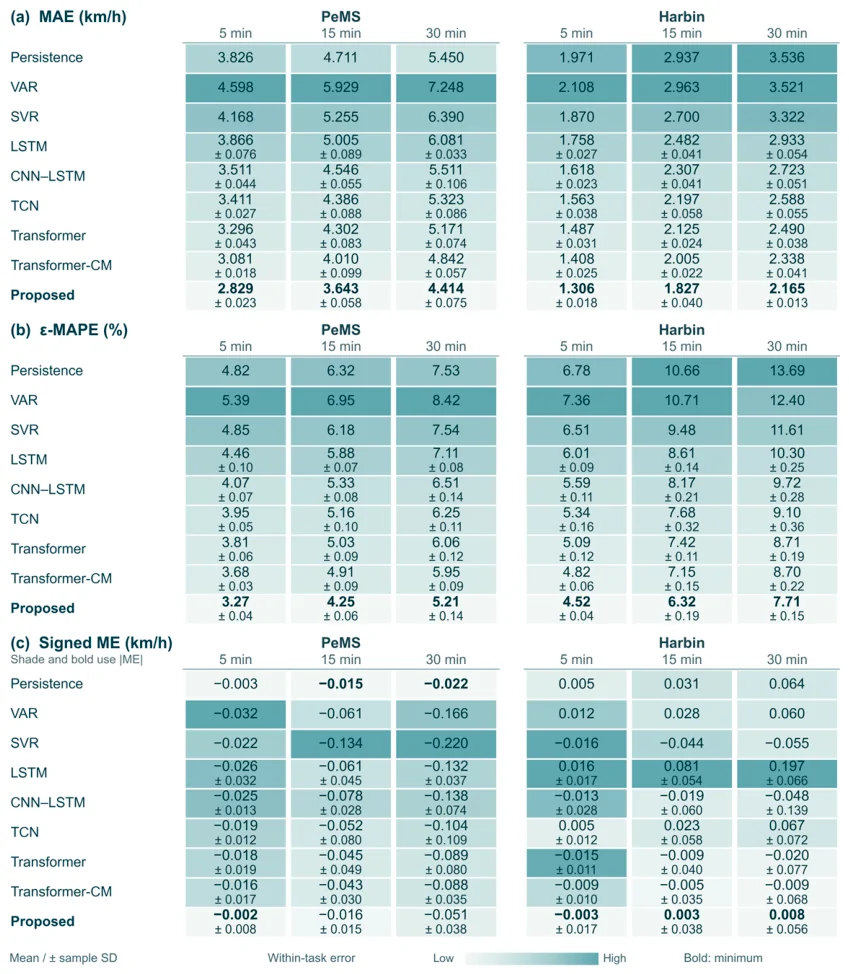
Three-location macro errors for nine methods on PeMS and Harbin at 5, 15 and 30 min: (**a**) mean absolute error (MAE, km/h), (**b**) denominator-bounded mean absolute percentage error (ε-MAPE, %; *ε* = 5 km/h), and (**c**) signed mean error (ME, km/h). For stochastic methods, cells give means with five-run sample standard deviations (SDs) below; deterministic methods have single values. Within each task column, darker shades indicate larger errors, using |ME| in (**c**); bold means identify the minimum error.

**Figure 10:**
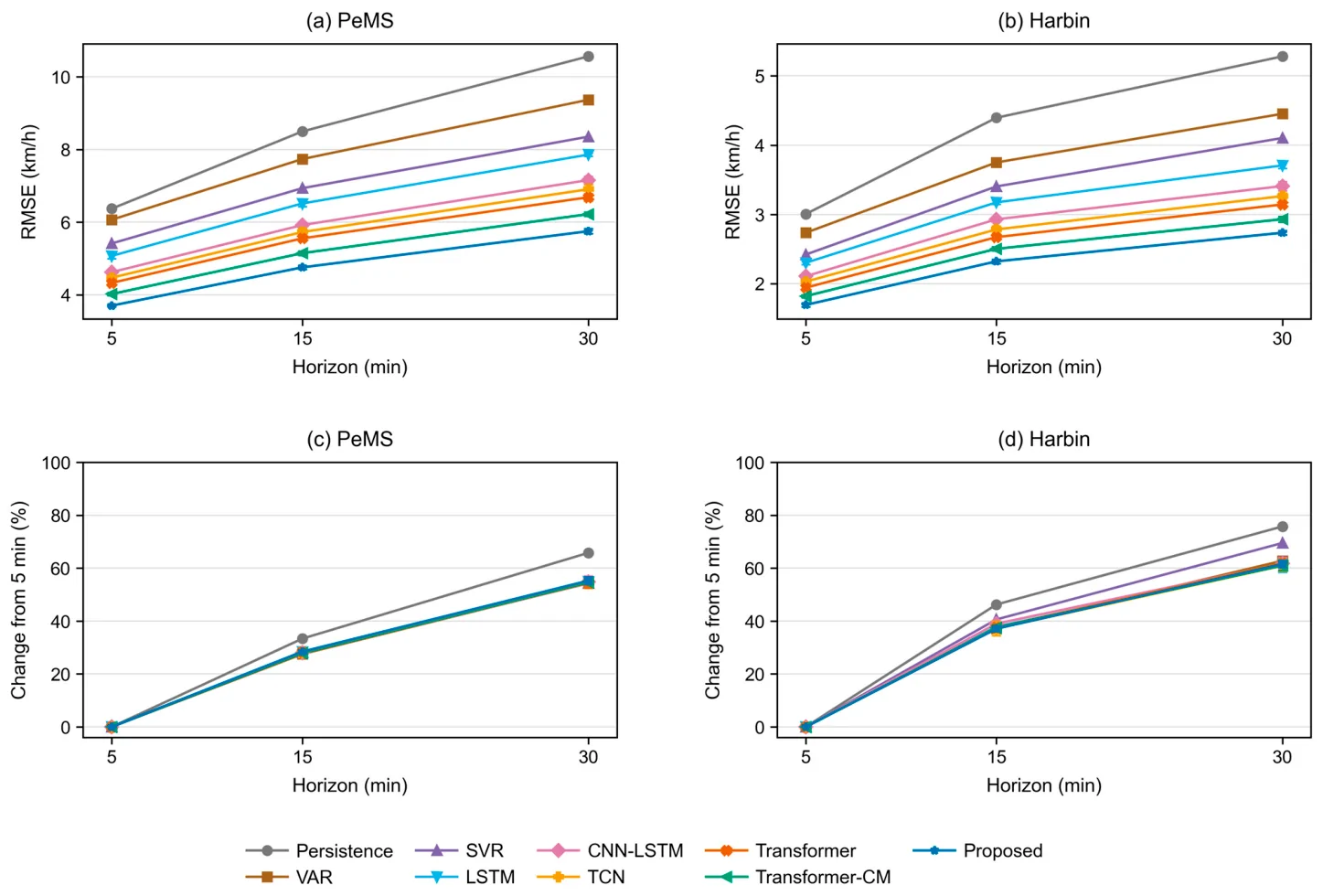
Test-set performance of all nine forecasting methods on (**a**,**c**) PeMS and (**b**,**d**) Harbin. The upper panels report equal-location macro root mean square error (RMSE), and the lower panels report the percentage RMSE increase relative to the 5 min horizon. For stochastic models, curves and error bars denote the mean and sample standard deviation over five fixed seeds. Persistence, VAR, and SVR each have one deterministic result and no standard-deviation bar. Individual seeded trajectories are omitted for clarity.

**Figure 11:**
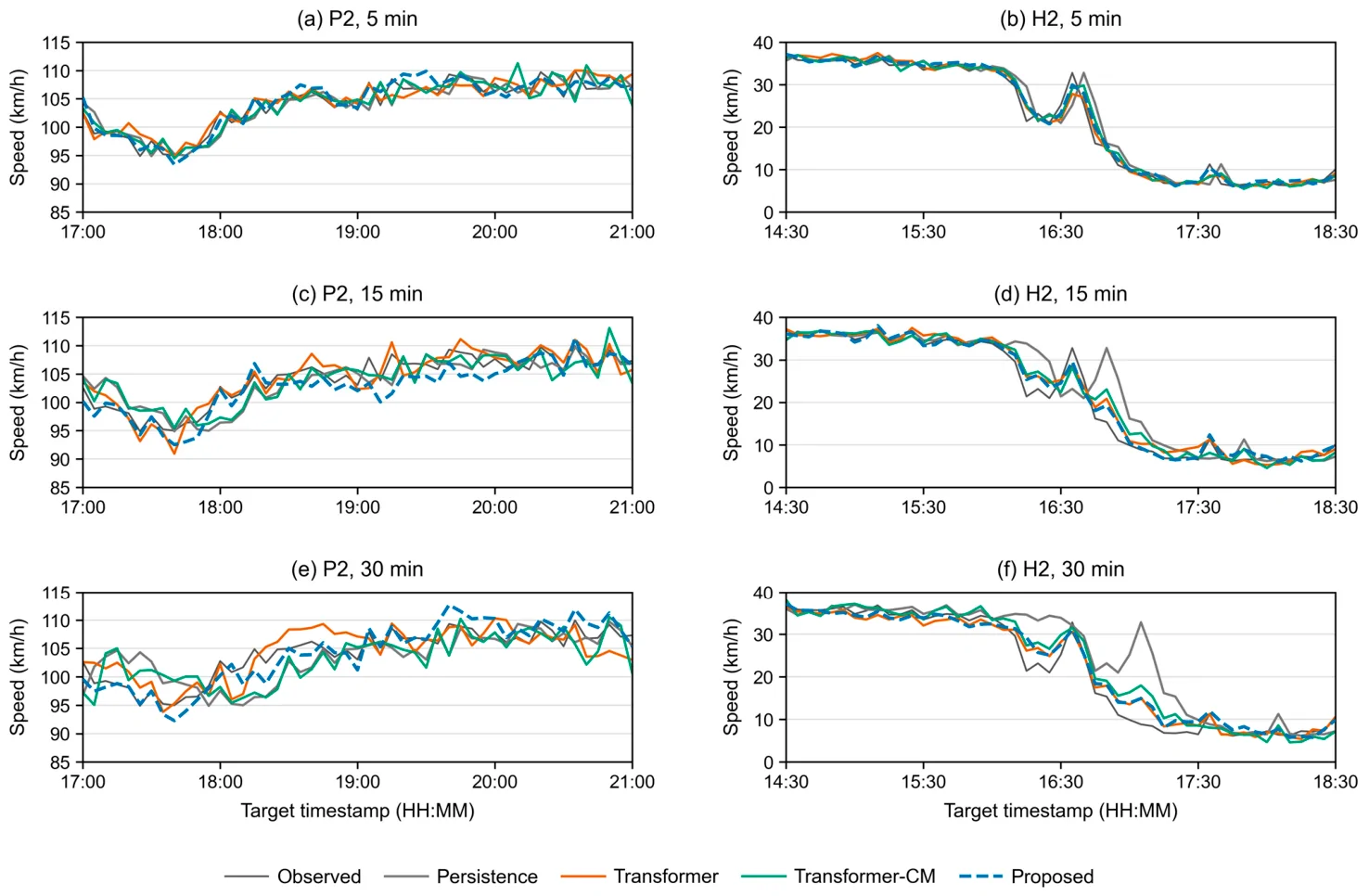
Four-hour test trajectories for freeway detector P2 on 27 December 2021, 17:00–21:00 (**a**,**c**,**e**), and Harbin Road segment H2 on 20 May 2022, 14:30–18:30 (**b**,**d**,**f**), at 5, 15, and 30 min horizons. Each panel retains 49 five-minute observations including both endpoints. Curves show observations, Persistence, Transformer, Transformer-CM, and the proposed method; stochastic predictions are averaged over five seeds.

**Figure 12:**
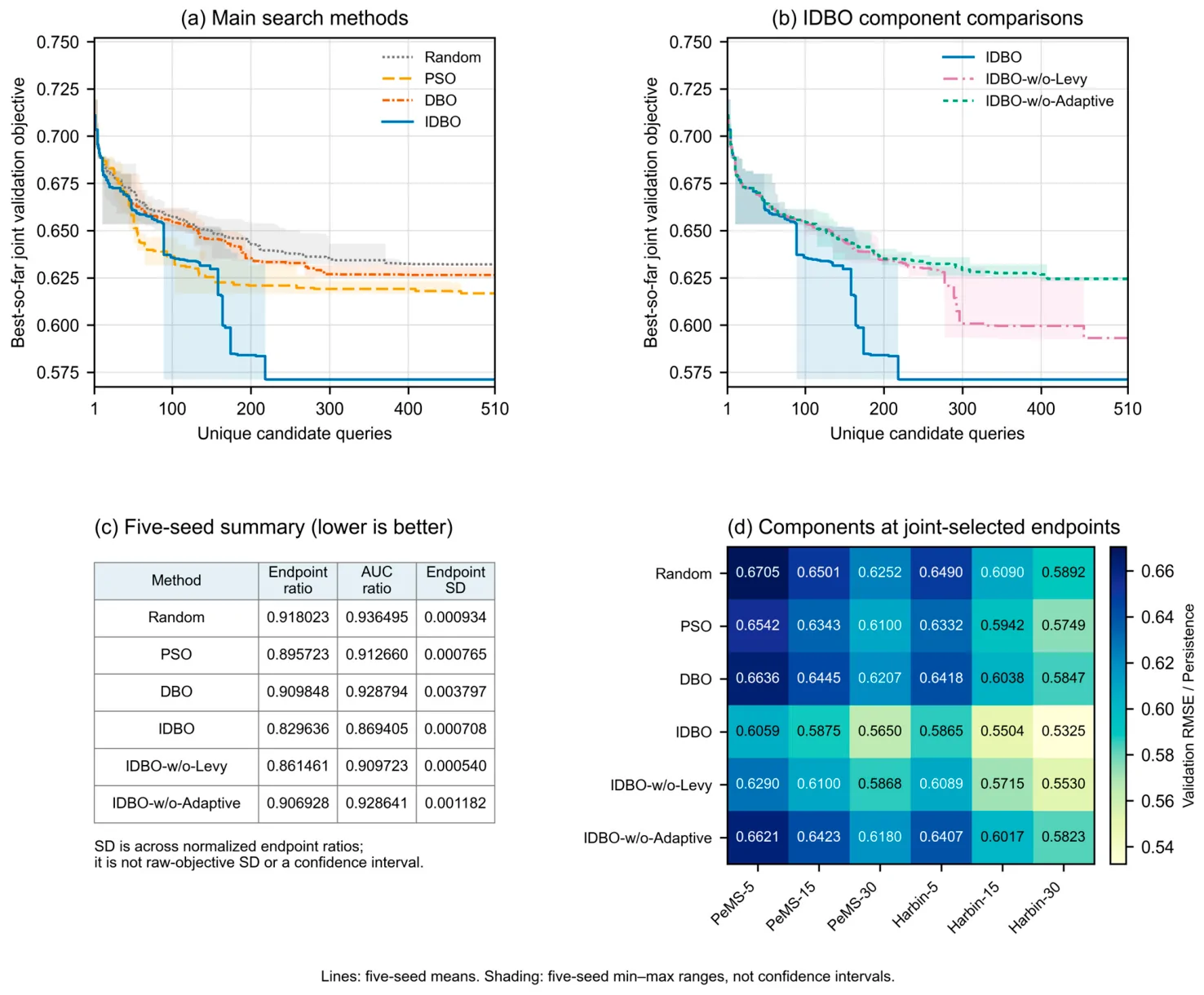
Dimensionless joint-validation objective over 510 unique configuration queries. (**a**) Random, PSO, DBO and IDBO; (**b**) IDBO and its two component controls. Lines show five-seed means of best-so-far histories; bands span their pointwise minima and maxima. (**c**) Mean normalized endpoint and area under the curve (AUC), with endpoint sample standard deviation (SD). (**d**) Validation RMSE/Persistence components at each run’s joint-selected endpoint, averaged across five seeds.

**Figure 13:**
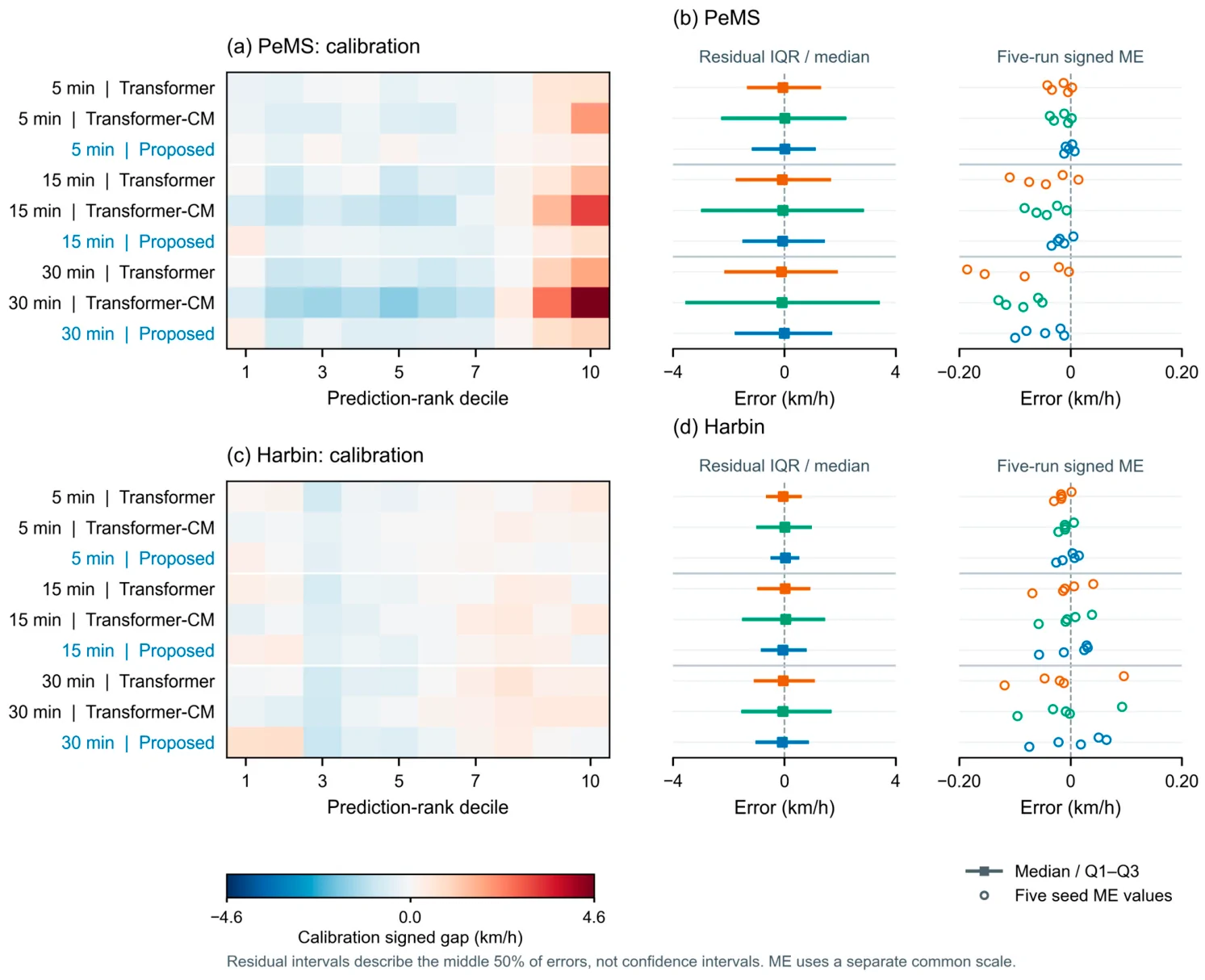
Calibration and residual diagnostics for Transformer, Transformer-CM and Proposed on PeMS (**a**,**b**) and Harbin (**c**,**d**), grouped by 5, 15 and 30 min horizon. Panels (**a**,**c**) show prediction-minus-observation gaps in ten method-specific prediction-rank bins. Panels (**b**,**d**) show residual medians and Q1–Q3 intervals of run-averaged predictions (**left**), and five run-level macro signed mean error (ME) values (**right**). Values are in km/h; each subcolumn uses a common scale across datasets. In (**b**,**d**), orange, green, and blue denote Transformer, Transformer-CM, and Proposed, respectively.

**Figure 14:**
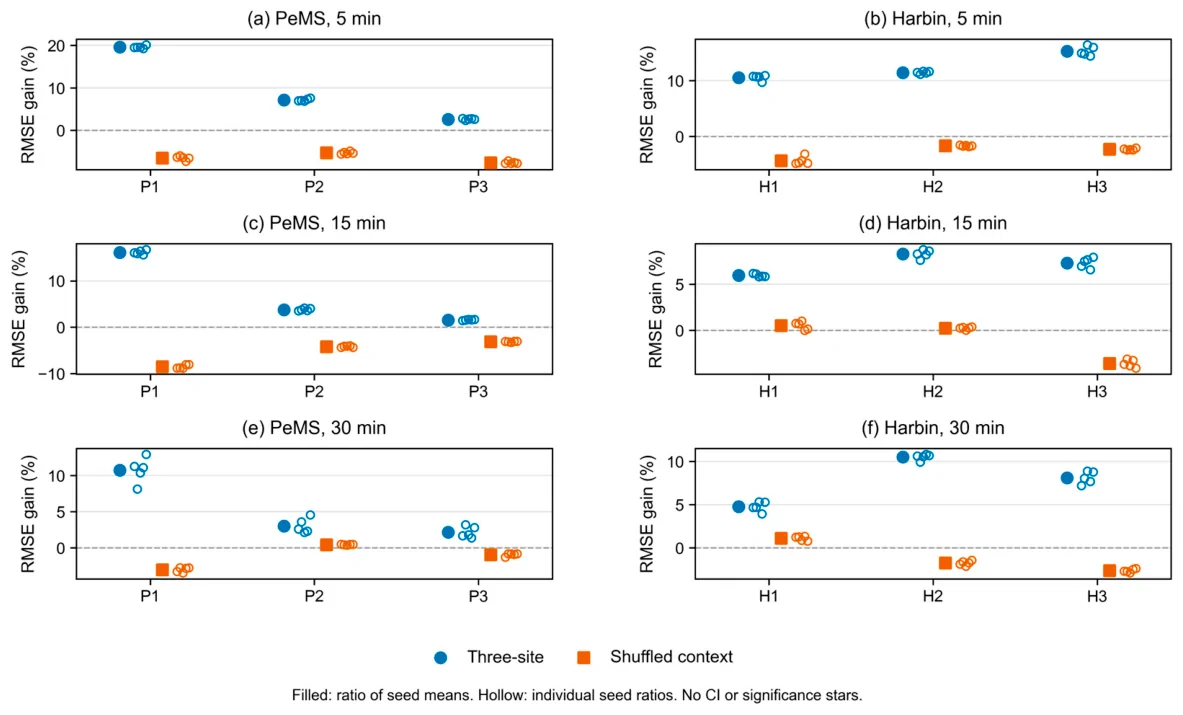
Location-level root mean square error (RMSE) gain of Three-site and Shuffled-context relative to Self-only for PeMS (**a**,**c**,**e**) and Harbin (**b**,**d**,**f**) at 5, 15, and 30 min. Filled markers denote percentage reductions computed from the five-run mean RMSE values; hollow markers show the five paired run-level percentage reductions. Positive values indicate improvement. The controls are descriptive comparisons without confidence intervals or significance markers.

**Figure 15:**
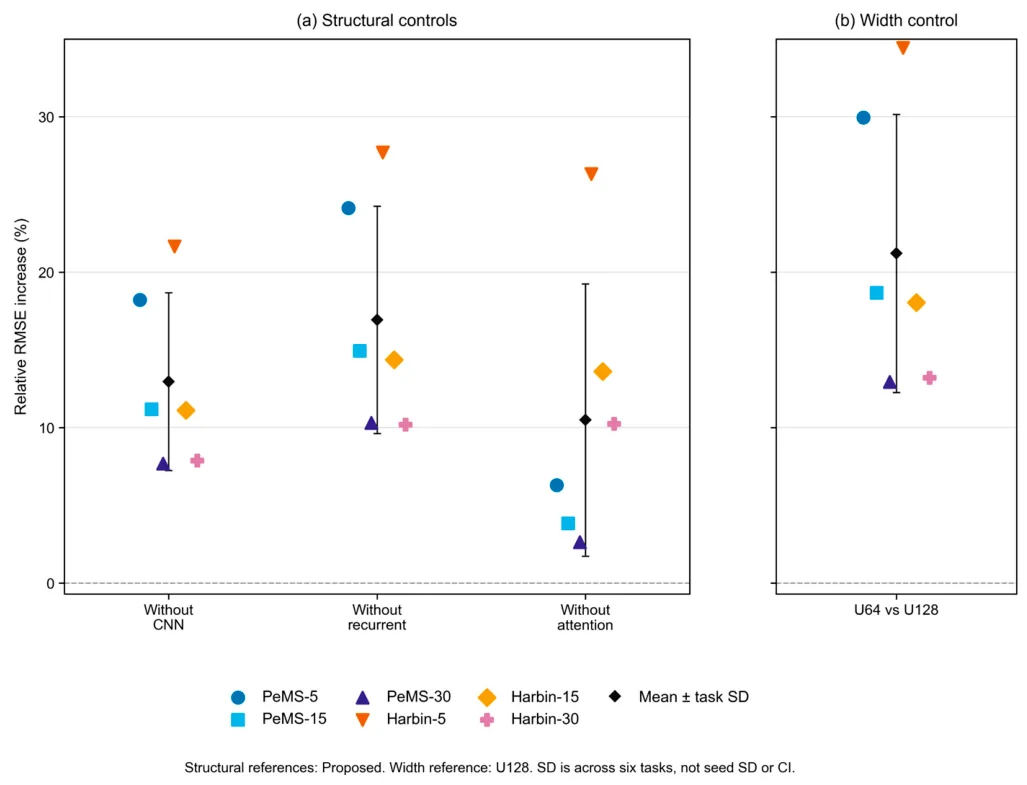
Relative root mean square error (RMSE) increase for (**a**) convolutional, recurrent, and temporal-attention removals relative to the full proposed configuration and (**b**) width U64 relative to U128, with U128 identical to the full proposed configuration. Each comparison shows all six dataset–horizon values and their equal-weight mean ± between-task sample standard deviation. Positive values indicate worse performance than the stated reference.

**Figure 16:**
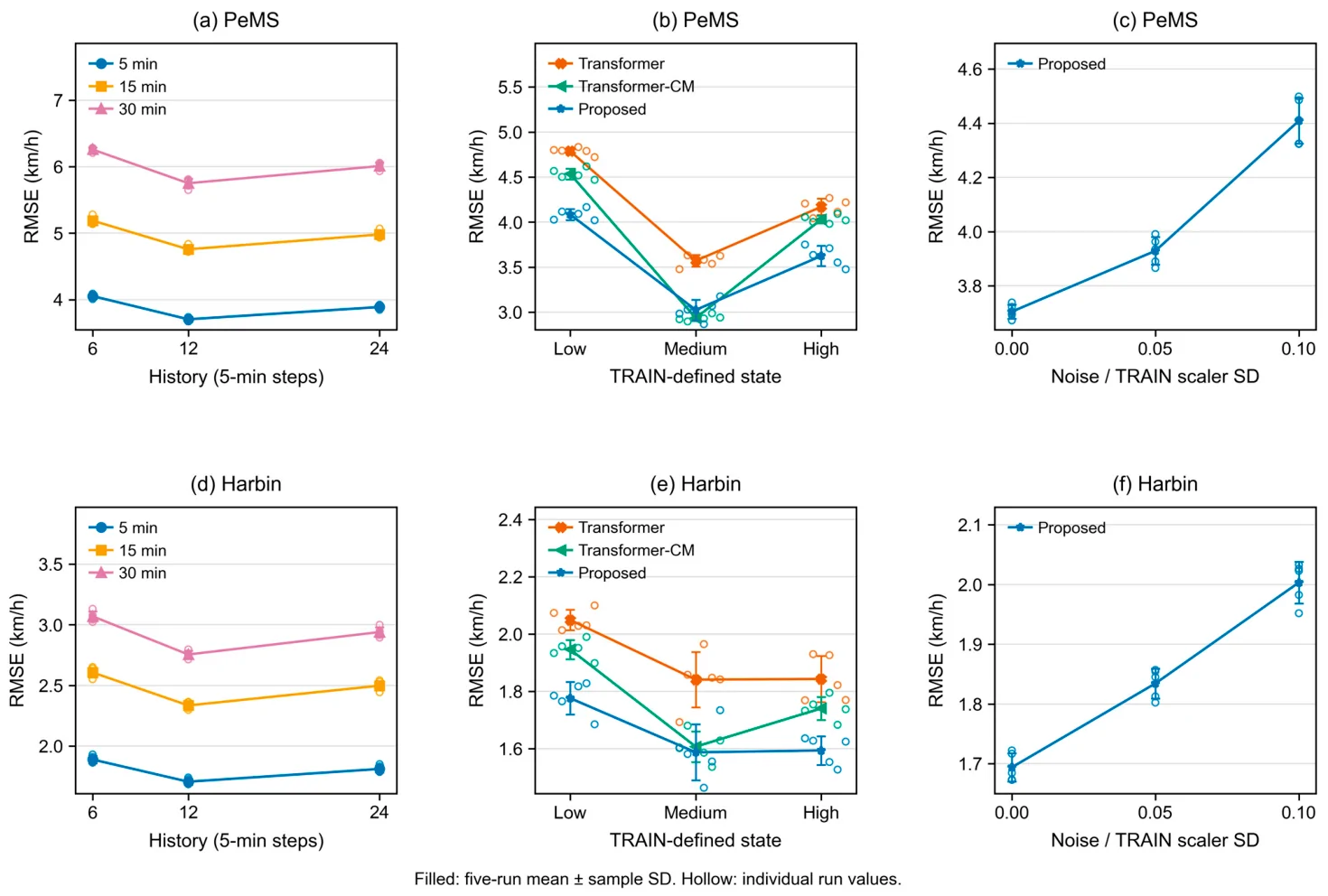
Input-window sensitivity, 5 min speed-state errors, and 5 min input-noise responses for the freeway (**a**–**c**) and urban-road (**d**–**f**) datasets. Filled markers show five-run mean root mean square error (RMSE) with ± one sample standard deviation; hollow markers retain all five run-level RMSE values.

**Table 2:** Input–output forms, core definitions and candidate domains, benchmark roles, and run counts for the nine forecasting methods. MACs denote multiply–accumulate operations.

Model	Category	Input → Output	Core Definition/Candidate Domain	Role	Runs
Persistence	Observed-history baseline	Latest available speed at each of three locations → 3 speeds	Repeats the last observation at the forecast issue time	Naïve no-learning reference	Deterministic single result
VAR	Linear multivariate baseline	12-step, three-location speed history → three speeds	Vector autoregression with intercept; lag *p* = {3, 6, 12}; ridge alpha = {0.01, 0.04, 0.16}; 9 candidates per task	Linear cross-location dependence	Deterministic single result
SVR	Kernel baseline	12-step, three-location speed history → three speeds	Three RBF regressors; *C* = {1, 10, 100}, gamma = {0.01, 0.1, 1}, epsilon = {0.05, 0.10, 0.20}; 27 candidates per task	Nonlinear shallow-learning reference	Deterministic single result
LSTM	Recurrent neural baseline	12-step, three-location speed history → three speeds	One sequence encoder with hidden units = {64, 96, 128, 160} and a linear three-output head; readout L2 = {0.01, 0.04, 0.16}; 12 candidates per task	Sequential dependence without CNN/TSAN	Five fixed seeds
CNN–LSTM	Hybrid neural baseline	12-step, three-location speed history → three speeds	Same-padded kernel-3 Conv1D, filters = {32, 64, 96}; LSTM units = {64, 128, 192}; linear three-output head; readout L2 = {0.01, 0.04, 0.16}; 27 candidates per task	Local plus recurrent representation	Five fixed seeds
TCN	Convolutional neural baseline	12-step, three-location speed history → three speeds	Input projection and three two-convolution residual blocks, kernel 3 and dilations {1, 2, 4}; channels = {16, 32, 48, 64}; readout L2 = {0.01, 0.04, 0.16}; 12 candidates per task	Non-recurrent long-range temporal reference	Five fixed seeds
Transformer	Attention neural baseline	12-step, three-location speed history → three speeds	Two temporal encoder layers; model dimension 64, four heads, feed-forward dimension 128 and Dense(3); readout L2 = {0.01, 0.04, 0.16}; 3 candidates per task	Temporal attention benchmark	Five fixed seeds
Transformer-CM	Capacity-matched attention baseline	12-step, three-location speed history → three speeds	Feed-forward width = 16:8:128; model dimension = 32:8:256; heads = {1, 2, 4, 8}; encoder depth = {1, 2, 3}; 5220 structures crossed with readout L2 = {0.01, 0.04, 0.16}	Parameter- and MAC-matched comparison; eligibility and task-wise selection in Section 4.3.2	Five fixed seeds
Proposed	IDBO–CNN–LSTM–TSAN	12-step, three-location speed history → three speeds	Same-padded kernel-3 Conv1D(*F*), LSTM(*U*), *A*-head TSAN with attention dimension *U* and feed-forward dimension 2*U*, Dense(64) and Dense(3); depth 1, readout L2 = 0.04; *F* = 16:8:128, *U* = 32:8:256, *A* = {1,2,4,8}; 1740 structures	Full synchronized three-location method	Five fixed seeds

Abbreviations: VAR, vector autoregression; SVR, support vector regression; RBF, radial basis function; LSTM, long short-term memory; CNN, convolutional neural network; TCN, temporal convolutional network; IDBO, Improved Dung Beetle Optimizer; TSAN, temporal self-attention network. CM denotes capacity matching.

**Table 3:** Shared experimental protocol and analytical complexity and efficiency estimates for the six dataset–horizon tasks. Structure quantities are single values where identical across tasks and otherwise task-wise ranges. Batch-one scenario values follow from the listed counts and the common calculation in Section 4.3.2. SVR complexity uses the declared support-slot proxy.

Group	Item/Model	Setting/Parameters (Count)	Budget/MACs per Sample	Note/FP32 Size (MiB)
Data	Input and targets	12 history steps; direct three-location output	1, 3, 6 steps = 5, 15, 30 min	One task per dataset–horizon pair
	Split and scaling	70%/20%/remainder provisional nominal-target boundaries	Training-fitted per-channel z-score and input imputation	Complete targets; common cross-horizon validation boundary; Table 1
Training	Final neural protocol	Adam; MSE with readout L2; learning rate 0.001; batch 32; dropout 0.2 where applicable	At most 30 epochs; patience 5	Restore best validation weights
	Stochastic candidate evaluation	Candidate seeds: 17, 29, 43	At most 15 epochs; patience 5	Equal-seed validation mean; deterministic candidates once
Reporting	Final prediction seeds	42, 2026, 3407, 7919, 10,007	Five final runs	Arithmetic mean and sample SD; deterministic baselines once
Statistics	Fixed comparator families	Transformer and Transformer-CM	6 DM–HLN tests per comparator	HAC lag *H*−1; within-family Holm; 12-point blocks; 5000 resamples
Search	Matched proposal budget	Random, PSO, DBO, IDBO, IDBO-w/o-Levy, IDBO-w/o-Adaptive	10 initial + 50 × 10 = 510 unique proposals	Same ordered initial ten within seed; duplicate repair before evaluation
	Search repetitions	Search seeds: 101, 503, 907, 1301, 1709	Five runs of the same joint six-task search	Endpoint/AUC relative to common initial best; endpoint sample SD
	Grid and shared selection	*F* = 16:8:128; *U* = 32:8:256; *A* = {1, 2, 4, 8}	1740 structures; one six-task objective	One configuration from the five IDBO proposal-set union: *F* = 64, *U* = 128, *A* = 4
Capacity matching	Transformer-CM qualification	Parameter ratio and MAC ratio each in [0.92, 1.08]	At least 8 eligible structures per reference	Filter structure first; validation then candidate ID, separately per task
Complexity	Persistence	0	0	0.000
	VAR	57	54	0.000
	SVR (support-slot proxy)	56,835	113,664	0.217
	LSTM	67,971	805,248	0.259
	CNN-LSTM	99,843	1,186,944	0.381
	TCN	42,099	499,536	0.161
	Transformer	67,395	825,792	0.257
	Transformer-CM	229,603	2,798,064	0.876
	Proposed	240,387	2,804,672	0.917
Efficiency	Batch-one cost scenario	Proposed scenario: median 37.662 ms; p95 44.441 ms	100 million MAC/s; 100 MB/s parameter transfer	Five common cost factors; p95 = 0.0148% of 300,000 ms

Abbreviations: MACs, multiply–accumulate operations; SD, standard deviation; DM–HLN, Diebold–Mariano test with the Harvey–Leybourne–Newbold correction; HAC, heteroskedasticity- and autocorrelation-consistent; MiB, mebibytes; FP32, 32-bit floating point. MSE denotes mean squared error. AUC denotes area under the curve, evaluated as the mean normalized best-so-far value over the 510 proposals.

**Table 4:** Equal-weight three-location macro root mean square error (RMSE, km/h) for nine methods on the Caltrans Performance Measurement System (PeMS) and Harbin datasets at 5, 15, and 30 min horizons; stochastic neural results are mean ± sample standard deviation over five runs, whereas deterministic baselines are single values.

Dataset	Model	5 Min Horizon	15 Min Horizon	30 Min Horizon	Runs
PeMS	Persistence	6.371	8.496	10.561	Single value
PeMS	VAR	6.067	7.737	9.367	Single value
PeMS	SVR	5.415	6.936	8.352	Single value
PeMS	LSTM	5.073 ± 0.080	6.515 ± 0.090	7.859 ± 0.040	5 runs
PeMS	CNN–LSTM	4.626 ± 0.040	5.921 ± 0.056	7.159 ± 0.101	5 runs
PeMS	TCN	4.473 ± 0.042	5.729 ± 0.090	6.907 ± 0.120	5 runs
PeMS	Transformer	4.329 ± 0.029	5.556 ± 0.080	6.686 ± 0.100	5 runs
PeMS	Transformer-CM	4.024 ± 0.045	5.148 ± 0.061	6.222 ± 0.056	5 runs
PeMS	Proposed	**3.705** ± 0.026	**4.758** ± 0.049	**5.752** ± 0.070	5 runs
Harbin	Persistence	3.006	4.396	5.282	Single value
Harbin	VAR	2.736	3.750	4.453	Single value
Harbin	SVR	2.421	3.404	4.103	Single value
Harbin	LSTM	2.302 ± 0.025	3.174 ± 0.029	3.707 ± 0.040	5 runs
Harbin	CNN–LSTM	2.108 ± 0.026	2.930 ± 0.035	3.410 ± 0.066	5 runs
Harbin	TCN	2.030 ± 0.031	2.784 ± 0.050	3.265 ± 0.043	5 runs
Harbin	Transformer	1.942 ± 0.021	2.672 ± 0.033	3.144 ± 0.044	5 runs
Harbin	Transformer-CM	1.821 ± 0.018	2.504 ± 0.028	2.931 ± 0.040	5 runs
Harbin	Proposed	**1.694** ± 0.024	**2.323** ± 0.030	**2.736** ± 0.032	5 runs

Note: Bold values indicate the lowest mean RMSE within each dataset–horizon comparison.

**Table 5:** Task-level Diebold–Mariano tests with the Harvey–Leybourne–Newbold correction (DM–HLN) and moving-block bootstrap estimates of macro RMSE gain relative to Transformer and Transformer-CM. Each comparator forms a separate six-task Holm family. *N* counts paired target timestamps after the common complete-target mask; location and seed losses are aggregated within timestamp. *p*-values are Holm-adjusted and 95% confidence intervals (CIs) are unadjusted; sig. and NS denote significant and non-significant decisions.

Dataset	Comparator	Forecast Steps	Paired Target Times (*N*)	DM–HLN	Holm *p* (Decision)	RMSE Gain [Unadjusted 95% CI] (%)
Harbin	Transformer	1	278	8.379	<0.001 (sig.)	12.79 [10.02, 14.82]
Harbin	Transformer-CM	1	278	4.187	<0.001 (sig.)	6.96 [2.46, 10.49]
Harbin	Transformer	3	276	7.288	<0.001 (sig.)	13.08 [10.01, 16.72]
Harbin	Transformer-CM	3	276	3.344	0.002 (sig.)	7.24 [3.79, 12.53]
Harbin	Transformer	6	273	5.563	<0.001 (sig.)	12.98 [8.50, 16.69]
Harbin	Transformer-CM	6	273	1.969	0.050 (sig.)	6.66 [0.76, 13.03]
PeMS	Transformer	1	1008	14.513	<0.001 (sig.)	14.42 [13.02, 15.76]
PeMS	Transformer-CM	1	1008	6.719	<0.001 (sig.)	7.92 [4.30, 10.99]
PeMS	Transformer	3	1007	13.432	<0.001 (sig.)	14.36 [12.43, 16.23]
PeMS	Transformer-CM	3	1007	4.868	<0.001 (sig.)	7.58 [3.16, 11.00]
PeMS	Transformer	6	1007	10.009	<0.001 (sig.)	13.97 [11.97, 16.01]
PeMS	Transformer-CM	6	1007	3.406	0.002 (sig.)	7.55 [2.83, 11.33]

## Data Availability

The PeMS data are accessible through the Caltrans Performance Measurement System (PeMS; https://pems.dot.ca.gov/, accessed on 31 July 2026). The Harbin Road-segment average speeds were obtained through the Baidu Maps traffic-data interface. Processed Harbin data and analysis materials supporting this study are available from the corresponding author on reasonable request, subject to the applicable data-provider terms.
